# Saliva microbiome in relation to SARS-CoV-2 infection in a prospective cohort of healthy US adults

**DOI:** 10.1016/j.ebiom.2023.104731

**Published:** 2023-07-22

**Authors:** Abigail J.S. Armstrong, Daniel B. Horton, Tracy Andrews, Patricia Greenberg, Jason Roy, Maria Laura Gennaro, Jeffrey L. Carson, Reynold A. Panettieri, Emily S. Barrett, Martin J. Blaser

**Affiliations:** a Center for Advanced Biotechnology and Medicine, Rutgers University, Piscataway, New Jersey, USA; b Department of Pediatrics, Rutgers Robert Wood Johnson Medical School, New Brunswick, New Jersey, USA; c Rutgers Center for Pharmacoepidemiology and Treatment Science, Institute for Health, Health Care Policy, and Aging Research, New Brunswick, New Jersey, USA; d Department of Biostatistics and Epidemiology, Rutgers School of Public Health, Piscataway, New Jersey, USA; e Department of Medicine, Public Health Research Institute, New Jersey Medical School, Rutgers University, Newark, New Jersey, USA; f Department of Medicine, Rutgers Robert Wood Johnson Medical School, New Brunswick, New Jersey, USA; g Environmental and Occupational Health Sciences Institute, Rutgers University, Piscataway, New Jersey, USA

**Keywords:** SARS-CoV-2, Microbiome, Saliva, Upper respiratory track

## Abstract

**Background:**

The clinical outcomes of SARS-CoV-2 infection vary in severity, potentially influenced by the resident human microbiota. There is limited consensus on conserved microbiome changes in response to SARS-CoV-2 infection, with many studies focusing on severely ill individuals. This study aimed to assess the variation in the upper respiratory tract microbiome using saliva specimens in a cohort of individuals with primarily mild to moderate disease.

**Methods:**

In early 2020, a cohort of 831 adults without known SARS-CoV-2 infection was followed over a six-month period to assess the occurrence and natural history of SARS-CoV-2 infection. From this cohort, 81 participants with a SARS-CoV-2 infection, along with 57 unexposed counterparts were selected with a total of 748 serial saliva samples were collected for analysis. Total bacterial abundance, composition, population structure, and gene function of the salivary microbiome were measured using *16S rRNA* gene and shotgun metagenomic sequencing.

**Findings:**

The salivary microbiome remained stable in unexposed individuals over the six-month study period, as evidenced by all measured metrics. Similarly, participants with mild to moderate SARS-CoV-2 infection showed microbiome stability throughout and after their infection. However, there were significant reductions in microbiome diversity among SARS-CoV-2-positive participants with severe symptoms early after infection. Over time, the microbiome diversity in these participants showed signs of recovery.

**Interpretation:**

These findings demonstrate the resilience of the salivary microbiome in relation to SARS-CoV-2 infection. Mild to moderate infections did not significantly disrupt the stability of the salivary microbiome, suggesting its ability to maintain its composition and function. However, severe SARS-CoV-2 infection was associated with temporary reductions in microbiome diversity, indicating the limits of microbiome resilience in the face of severe infection.

**Funding:**

This project was supported in part by Danone North America and grants from the 10.13039/100000002National Institutes of Health, United States.

**Research in context**

**Evidence before this study**

Prior research has investigated the association between the human microbiome and the clinical outcomes of SARS-CoV-2 infection. However, many studies have focused on the gut microbiome with the specific impact of SARS-CoV-2 infection on the salivary microbiome and its relationship with disease severity having been less explored. While existing studies have provided insights into the stability and resilience of the microbiome in various health conditions, there is a gap in knowledge regarding the changes occurring in the salivary microbiome during different stages of SARS-CoV-2 infection.

**Added value of this study**

This study contributes to the existing knowledge by examining the specific changes in the salivary microbiome during different stages of SARS-CoV-2 infection. The study utilized saliva samples collected before, during, and after infection to comprehensively analyze the total bacterial abundance, composition, population structure, and gene function of the microbiome throughout the course of infection. A unique feature of this study are the unexposed samples prior to any SARS-CoV-2 infection which provide insights into microbiome changes during infection relative to before.

**Implications of all the available evidence**

This study demonstrated the relative stability of the salivary microbiome in mild to moderate SARS-CoV-2 infections with severe infections leading to significant reductions in microbiome diversity early after infection. These findings shed light on the salivary microbiome's resilience and limitations in the context of severe infection. This research contributes to the broader understanding of the complex interactions between the human microbiome and viral infections, potentially guiding future investigations on microbiome-based therapies or interventions for managing COVID-19.

## Introduction

Since late 2019, severe acute respiratory syndrome coronavirus 2 (SARS-CoV-2) has caused a worldwide pandemic that has sickened hundreds of millions of people with the illness called coronavirus disease of 2019 (COVID-19) and has killed millions.^1^ The severity of the infection has varied across the full range from asymptomatic carriage to death.^2^ Although numerous host factors affecting this clinical variation have been described, together they only account for a portion of the variation.^3^, ^4^, ^5^, ^6^, ^7^

The microbiome, the microbial populations living in and on the human body in enormous number in multiple discrete niches,^8^ shows extensive interpersonal heterogeneity in community structure and composition.^9^ Importantly, a healthy microbiome helps defend against a broad range of bacterial, viral, and eukaryotic pathogens.^10^, ^11^, ^12^ Conversely, these infections can directly disrupt the microbiome,^12^^,^^13^ and medical treatments, such as antibiotics, directed against pathogens, can cause further disruptions.^14^ For these reasons, studying the interactions between SARS-CoV-2 infection and the human microbiome is important, and there have been a number of prior studies, largely focused on the gut microbiome.^15^, ^16^, ^17^, ^18^, ^19^, ^20^, ^21^, ^22^

We identified three key questions about the interaction of SARS-CoV-2 infection and the human microbiome: (i) Does the nature of the pre-existing microbiome predict the clinical intensity of the SARS-CoV-2 infection? (ii) How does the infection affect the community structure and composition of the microbiome? (iii) Is the severity of infection an important variable in any observed changes in the microbiome?

To directly address these questions, we leveraged a unique set of serial saliva specimens from a prospective study focused on the susceptibility of healthcare workers (HCW) and other university employees to SARS-CoV-2 in the first wave of COVID-19 in the United States, prior to the availability of vaccination.^23^^,^^24^ Oral microbiota also contribute to the lung microbiome,^25^, ^26^, ^27^ and their presence has been associated with inflammation.^26^ As such, the salivary microbiome not only provides an easily accessible proxy of microbes found in the more distal airways, but is also a potential indicator of lung health especially pertinent to those infected with SARS-CoV-2. We now report that in a SARS-CoV-2-naïve ambulatory population early in the pandemic, the oral microbiome was highly resilient in the face of the acute, generally mild or asymptomatic infection but shows alterations following illness in those with more severe symptoms.

## Methods

### Ethics statement

All study activities were approved by the Rutgers Institutional Review Board (Pro2020000679) and all participants provided electronic informed consent prior to engaging in study activities.

### Study cohort

**Fig. 1:**
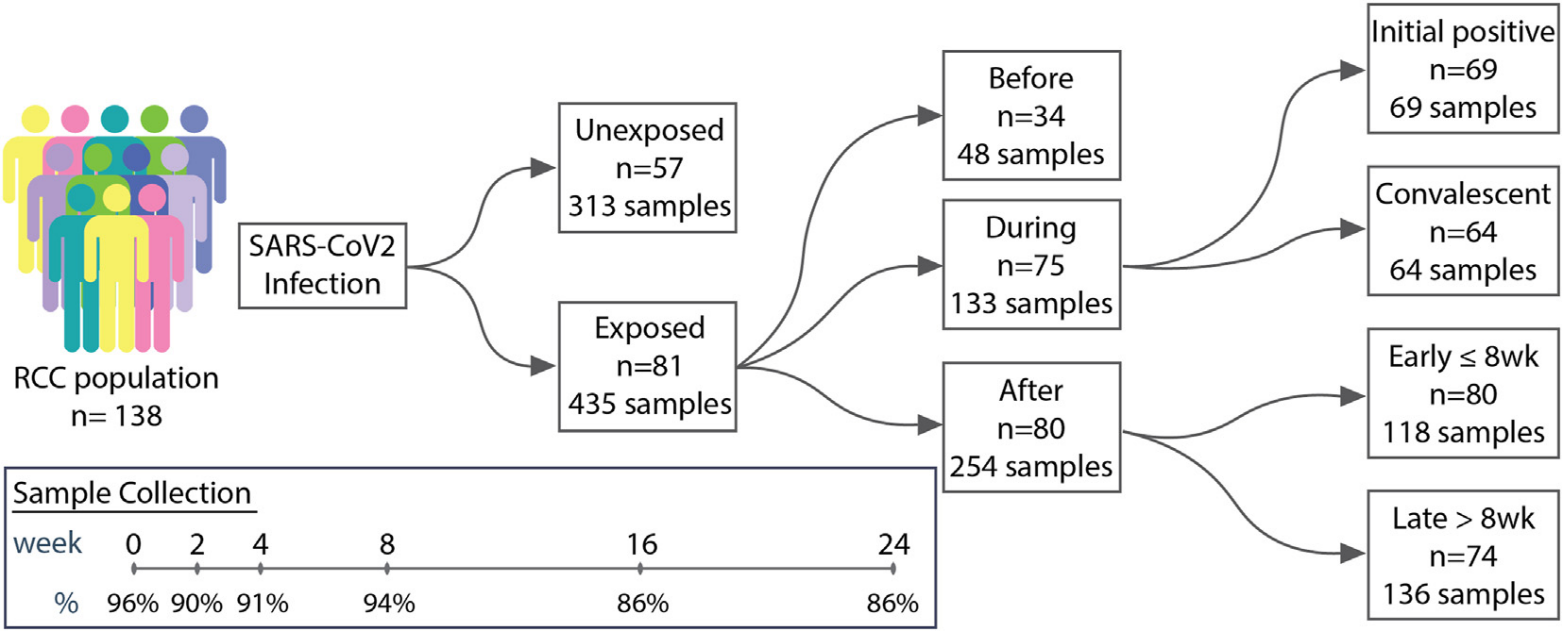
**Study design schematic.** Study participants are a subset from the larger population (n = 829) of the Rutgers Corona Cohort (RCC).^1^ COVID-negative subjects were selected from a larger pool of negative participants and matched 2:1 with the cases based on age, sex, BMI, and presence of co-morbidities.

Study participants represented a nested sample of SARS-CoV-2-exposed and unexposed persons from the Rutgers Corona Cohort (RCC), which prospectively examined the risk of acquiring SARS-CoV-2 infection in 548 healthcare workers (HCW), and 283 non-HCW employed by Rutgers University. This was an unvaccinated, immunologically naïve population in early 2020.^23^^,^^24^ Saliva samples were collected at intake (week 0) and at weeks 2, 4, 8, 16, and 24 (Fig. 1). SARS-CoV-2-infected participants were those with either: (i) a positive qPCR test from the collected saliva at any point and/or (ii) a person who had a positive serology for SARS-CoV-2 by ELISA, as described.^23^^,^^24^ For those who were seropositive only, the saliva sample from the same timepoint was considered as the closest indication of the time of infection. Since there was no prior literature reporting 6-month longitudinal studies of the salivary microbiome in healthy adults, we estimated that a 50% match would be sufficient to control for background variation. Unexposed participants were selected from the pool of unexposed participants during weeks 0–24 and matched to the exposed participants with a 1:2 ratio based on age group (by decade of life), sex, racial/ethnic category, and health care worker status (HCW or non-HCW). If matching based on all criteria was not feasible, the requirement for matching by HCW status was relaxed. If matching based on decade of life was not feasible, subjects were matched to comparators within ±10 years in age. Using this approach, exposed and unexposed groups were well-balanced by matched criteria as well as non-matched criteria, including BMI, GFR, and comorbidities such as cardiovascular disease and diabetes mellitus (Supplemental Material S1).

To account for participants becoming infected at different study weeks, we established a standardized reference point by defining the first positive sample for all exposed individuals as week 0, with all prior and subsequent samples shifted accordingly for those participants who became positive after the first study visit. For the unexposed participants, the weeks corresponding to their samples were shifted according to that of their matched exposed counterparts. Furthermore, to ensure completeness of data and to minimize missing data in analyses from those without samples prior to infection, we divided the subjects into two subcohorts(1): the During and After (DA) cohort comprised of exposed individuals who were positive at the first visit, together with their corresponding matched unexposed counterparts and(2) the Before, During, and After (BDA) cohort comprised of exposed individuals who were negative at their first visit and became positive at a subsequent visit 2, 4, or 8 weeks later and had a follow-up visit, together with their corresponding matched unexposed counterparts (Table S1). Sensitivity analysis was not performed on these subcohorts.

### Sample collection

Saliva samples were collected in person at every study visit using the Spectrum sDNA-1000 kit which includes a sample preservative and were stored in −80 °C. Per Rutgers University biosafety standards for working with samples that could potentially contain live SARS-CoV-2, all collected saliva samples were heat-decontaminated at 56 °C for 60 min. This decontamination step was performed after the samples were removed from storage at −80 °C and thawed and before any further handling.

### Outcome measures

The primary outcome measure for this study is the composition of the saliva microbiome as assessed by analysis of salivary DNA, which was quantified using multiple approaches. First, *16S rRNA* gene abundance was measured using quantitative polymerase chain reaction (qPCR). Second, the microbiome composition was assessed through 16S rRNA gene sequencing, which allowed for the measurement of taxonomic composition based on amplicon sequence variants (ASVs).^28^ To characterize the composition of the ASVs, alpha diversity metrics including Faith's Phylogenetic Diversity,^29^ Pielou Evenness,^30^ and number of observed features (ASVs)^28^ were calculated. Beta diversity metrics, specifically weighted and unweighted UniFrac,^31^ were used to evaluate the dissimilarity between samples. Moreover, the ASVs were further categorized at the phylum, genus, and species levels before statistical analysis.^32^ Lastly, the functional gene pathways of the microbiome were measured using shotgun metagenomic sequencing. To characterize the features obtained from shotgun metagenomic sequencing, the alpha diversity metrics Shannon entropy,^33^ Pielou Evenness, and observed features (gene pathways)^34^ were computed. The beta diversity metrics, Jaccard^35^ and Bray–Curtis^36^ analyses, were used to assess the dissimilarity between samples based on their functional gene pathways. In addition to the primary outcomes, a secondary outcome measured in the shotgun metagenomic sequencing was the sequencing depth, expressed as the number of gigabases, both in terms of total bases and the portion specifically mapping to bacterial reads.^34^

### DNA extraction

DNA was extracted using the DNeasy PowerSoil Kit protocol (Qiagen) for saliva, as described.^37^ Post-extraction DNA quantity and quality was measured using Thermo NanoDrop 1000.

### *16S rRNA* gene quantification

*16S rRNA* gene was quantified with qPCR using SYBR green following kit instructions on a Roche LightCycler 480. The total reaction volume in each well was 20 μL containing 10 μL of SYBR green master mix, 2 μL of sample, 0.5 μL each of forward and reverse primers, and 7 μL of RNase-free water. The samples were run with pre-incubation at 95 °C for 5 min and 45 amplification cycles of 95 °C for 10 s, 60 °C for 16 S, and 72 °C for 10 s, followed by a 1-min melting curve at 65 °C. The forward primer: 338F—ACTCCTACGGGAGGCAGCAG, and reverse primer: 518R–ATTACCGCGGCTGCTGG, were used.

### *16S rRNA* sequencing library preparation and sequencing

The *16S rRNA* gene sequencing library was prepared using the Earth Microbiome Project (EMP) protocol, as described.^37^^,^^38^ In short, extracted saliva DNA was PCR-amplified with barcoded primers targeting the V4 region of the *16S rRNA* gene according to the EMP 16S Illumina Amplicon protocol with the 515F:806R primer pairs. Control PBS and sample preservative that had undergone the same DNA extraction were also processed. Each PCR product was quantified using PicoGreen (Invitrogen), and equal amounts of DNA from each sample were pooled and cleaned using the UltraClean PCR Clean-Up Kit (MoBio) and assayed on one of two sequencing runs. DNA sequencing reactions were conducted at GENEWIZ, Inc. (South Plainfield NJ, USA) using a MiSeq sequencing platform (Illumina, San Diego CA, USA). We performed 2 × 250 sequencing. After sequencing, there were a total of 1.68E7 read pairs across both runs with a mean of 19,049 read pairs per sample. Post-processing, there was a total of 1.37E7 single forward reads with median of 15,756 (SD: 6185) single reads per sample. Samples with fewer than 3222 reads were excluded from the analysis.

### *16S rRNA* gene sequencing bioinformatics and statistical analysis

Data were processed using QIIME 2 version 2021.4.^39^ We performed the analysis using a single end processing pipeline. In short, sequences were demultiplexed/denoised using the DADA2 q2 plugin.^28^ Features were classified using skLearn implemented in QIIME2 with a classifier that was pre-trained on Silva 138. The phylogenetic tree was built using the SEPP plugin and the Silva 128 reference tree.^40^ Features that did not classify at the phylum level or were classified as mitochondria or chloroplast were filtered from the analysis. Samples were rarefied at 3222 reads. Diversity metrics and PERMANOVA statistical tests were calculated in QIIME2. Where reported as normalized values, alpha-diversity metrics were normalized as a proportion of the baseline. Specifically, each value was calculated as the ratio of that timepoint measurement in relation to the corresponding baseline timepoint for that subject. All other statistics were calculated using the programming language R. To account for the repeat measures on participants (across time), linear mixed effect models using the package nlme^41^ in R were used. Models included timepoint to account for variation over time and age group. When appropriate, we used Kruskal–Wallis rank sum test with Dunn's post-hoc test as necessary or Mann–Whitney U test using base R. All visualizations were created using ggplot2.^42^ Differential abundance tests were performed using MaAslin2^43^ with FDR correction. Features with a corrected q-value <0.05 were identified as significant.

### Shotgun metagenomic sequencing

Human DNA was depleted as described.^37^^,^^44^ In short, 250 μL from each saliva sample was centrifuged at 10,000 rcf at 4 °C for 9 min. Supernatant was discarded and pellets were resuspended in 200 μL of sterile H_2_0 and incubated at room temperature for 5 min, then 10 μL of a 0.2 mM PMA solution was added and samples were incubated in the dark for 5 min. Samples were then placed horizontally on ice <20 cm from a fluorescent light bulb (200 W, 2030 lumens, 3000K) for 25 min, vertexing and rotating every 5 min. Samples were stored at −20 °C until DNA extraction using the Qiagen PowerSoil kit, as described above.

DNA library preparations and sequencing reactions were conducted at Novogene Corporation Inc. The Illumina DNA Prep Kit was used to prepare the libraries according to the manufacturer's recommendations. The Qubit® 2.0 Fluorometer was used to measure library concentration, and size was measured via LabChip GX Chip using the DNA NGS 3K Assay Kit (PerkinElmer, Inc.). If necessary, samples were rerun using the BioAnalyzer 2100 (Agilent) with the Agilent High-Sensitivity DNA Kit. Libraries were quantified using QPCR with the KAPA SYBER FAST Library-Quant-Illumina Kit. Sequencing was performed using 150 paired end (PE150) configuration on the NovaSeq 6000 S4 Flowcell using Workflow A, with 1% PhiX DNA spiked in with pooled libraries as a control. The raw data from the Illumina platform are transformed to Sequenced Reads (Raw Data) by base calling. Data were checked for quality by analyzing the Distribution of Sequencing Quality (Q30 > 80%), the Distribution of Sequencing Error Rate, and the Distribution of A/T/G/C Bases. Data were filtered by removing reads containing N >10% (N representing bases that cannot be determined) and those reads containing low quality (Qscore ≤ 5) bases >50% of the total base count. After sequencing, there was a total of 2377.3 GB with a median of 18.3 GB per sample (IQR: 5.2) (Fig. S2). Post-processing, there was a total of 642.0 microbial GB with a median of 4.1 microbial GB per sample (IQR: 5.6), with a median of 24% of sequencing reads assigned to bacteria (Fig. S2).

### Shotgun bioinformatics and statistical analysis

Sequences were trimmed and human reads were removed using the tool Kneaddata v0.10.0 (https://github.com/biobakery/kneaddata) using default parameters. Gene function and pathway analysis was conducted using HuMAnN3 v3.0.0a4,^34^ with default parameters. Abundances of genes or pathways were renormalized to counts per million reads (CPM) using the HuMAnN3 utility script. Taxonomic identification was determined using MetaPhlAn3 v3.0.7^45^ with default parameters. Co-ocurrence networks were created using Sparse Correlation Network Investigation for Compositional data (SCNIC)^46^ with default parameters and a correlation significance being defined at a correlation co-efficient cutoff of >0.35. Differential abundance tests were performed using MaAslin2^43^ with FDR correction. Features with a corrected q-value <0.05 were identified as significant. When appropriate, we used the Kruskal–Wallis rank sum test with Dunn's post-hoc test as necessary or Mann–Whitney U test using base R.

### Role of funders

This project was supported in part by Danone North America (MJB) and grants from the National Institutes of Health: grant U01 AI122285 (MJB), R01AI158911 (MJB, EB. MLG, DBH), and R61HD105619 (DBH).

## Results

### Cohort description

Study participants were followed over 24 weeks and monitored for SARS-CoV-2 infection (Fig. 1). The SARS-CoV-2 positive participants (exposed group) were matched 2:1 based on age, sex, racial/ethnic category, and health care worker status (HCW or non-HCW) with unexposed individuals from the larger cohort who were never positive in the same time period (Table S1). Because the exposed group became positive at different times, we divided the subjects into two cohorts to avoid gaps in datapoints prior to infection: (1), those who were positive at the first visit (and their matched unexposed counterparts) were classified as the During and After (DA) cohort; and (2), those who were negative at their first visit and became positive at a subsequent visit 2, 4, or 8 weeks later (and their matched unexposed counterparts) were classified as the Before, During, and After (BDA) cohort (Fig. 1, Table S1). In this racially diverse cohort, most participants were women (73.2%), <60 years old (86.2%), not current smokers (95.7%), and 119 of the 138 participants (86.2%) were HCWs (Table S1). Of those with a SARS-CoV-2 infection during the 24-week study period, the majority reported no, mild, or moderate symptoms (71.6%) with only seven (8.6%) visiting the ER and three (3.7%) being admitted to the hospital none of whom were admitted to the ICU nor intubated (Table S1). There were minimal interventions in the SARS-CoV-2-exposed participants: five SARS-CoV-2-positive participants (6.1%) were treated with antibiotics and no unexposed individuals reported antibiotic use over the course of the study, one exposed participant (1.2%) received corticosteroids, two (2.5%) received anticoagulants, six (7.4%) received hydroxychloroquine, and one (1.2%) received the antiviral, remdesivir (Table S1). Participants were ill for a median of 17 ± 121 (IQR) days, with a bimodal distribution, almost entirely ≤30 or >90 days (Fig. S1).

### Total bacterial abundance

**Fig. 2:**
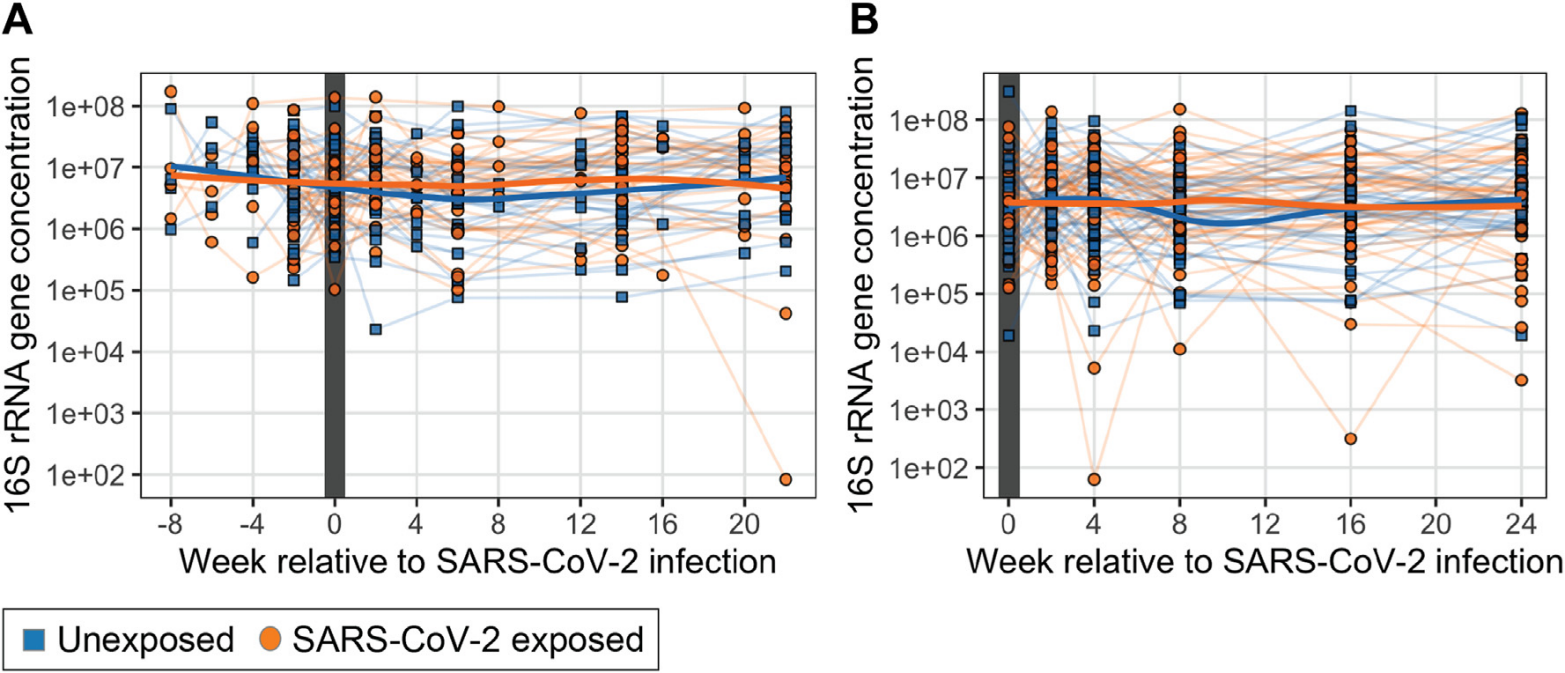
**Total bacterial populations in the saliva samples in relation to the baseline sample, based on enumeration of 16S rRNA gene copies.A.** SARS-CoV-2-infected subjects before, during, and after viral positivity (n = 34) and their 31 matched unexposed counterparts, with the study week shown relative to COVID infection with the week of the first positive test defined as week 0. **B.** SARs-CoV-2-infected subjects who were virus-positive at the first study visit (n = 47) and their 41 matched unexposed counterparts. There were no significant differences in either analysis between the exposed or unexposed subjects (Student's T test, FDR-corrected p > 0.05).

First, we investigated whether or not acute SARS-CoV-2 infection affected the bacteria concentrations in saliva. As expected, the bacterial concentrations did not vary significantly over time in the unexposed group (n = 57, linear mixed effects model, p > 0.05). In the 34 SARS-CoV-2 exposed participants from whom we could analyze specimens before, during, and after infection, we found no evidence of significant changes in bacterial abundance over time based on fitting linear mixed effects models accounting for time and repeat measures (Fig. 2A, p > 0.05). Similarly, in the 37 participants we followed after SARS-CoV-2 infection, there were no significant changes over time (Fig. 2B; linear mixed effects model, p > 0.05). Finally, comparing the specimens from all participants without SARS-CoV-2 infection to those from the 75 samples from participants during their infection, there was no significant difference in 16 S rRNA gene number across all timepoints (Student's T-test, p > 0.05). From these data, we concluded that acute SARS-CoV-2 infection did not significantly affect the oral bacterial load detected in expectorated saliva nor was there a significant difference in bacterial load throughout course of SARS-CoV-2 infection.

### 16S *rRNA* gene sequencing does not show significant changes in community richness and evenness over time in SARS-CoV-2 infection relative to uninfected participants

**Fig. 3:**
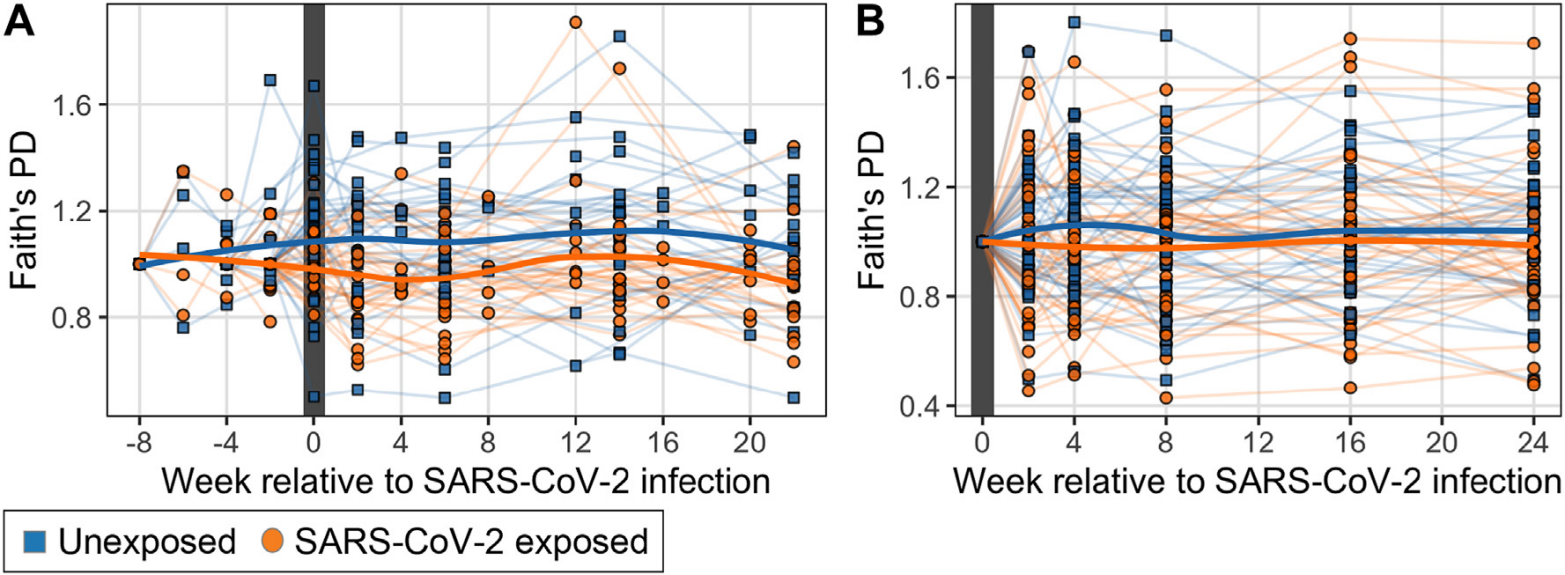
**Normalized alpha diversity of the salivary microbiome over time.** Alpha diversity as measured by Faith's phylogenetic diversity (PD) normalized based on proportion of the first timepoint for each individual. **A.** SARS-CoV-2 infected subjects (n = 34) before, during, and after viral positivity and their 31 matched unexposed counterparts, with the study week shown relative to COVID infection with the week of the first positive test defined as week 0. **B.** SARS-CoV-2-infected subjects (n = 47) who were virus-positive at the first study visit and their 41 matched unexposed counterparts. There were no significant differences in either analysis between exposed and unexposed subjects (p > 0.05) when controlling for age and week of sampling, using linear mixed effects modeling.

To examine how the diversity of the salivary microbiota changes in relation to SARS-CoV-2 infection, we modeled alpha diversity over time controlling for infection status and age. To better examine relative change within each participant over time, we normalized the data at every timepoint as a proportion of the baseline (Fig. 3, Fig. S2); we also conducted the same analyses without normalization (Fig. S2). In each of these analyses of both richness and evenness, we found that alpha diversity did not significantly differ between the SARS-CoV-2 exposed and unexposed participants either throughout the course of SARS-CoV-2 infection (BDA sub-cohort) or in recovery after SARS-CoV-2 infection (DA sub-cohort) (Fig. 3, Fig. S2; linear mixed effects model, p > 0.05). Thus, in this group of mostly symptomatic participants with clinically mild or moderate infections, who had minimal or no antibiotic exposure, their SARS-CoV-2 infection did not substantially affect the alpha diversity of the bacterial populations in saliva.

### Effect of SARS-CoV-2 infection on community composition of the salivary bacterial populations

**Fig. 4:**
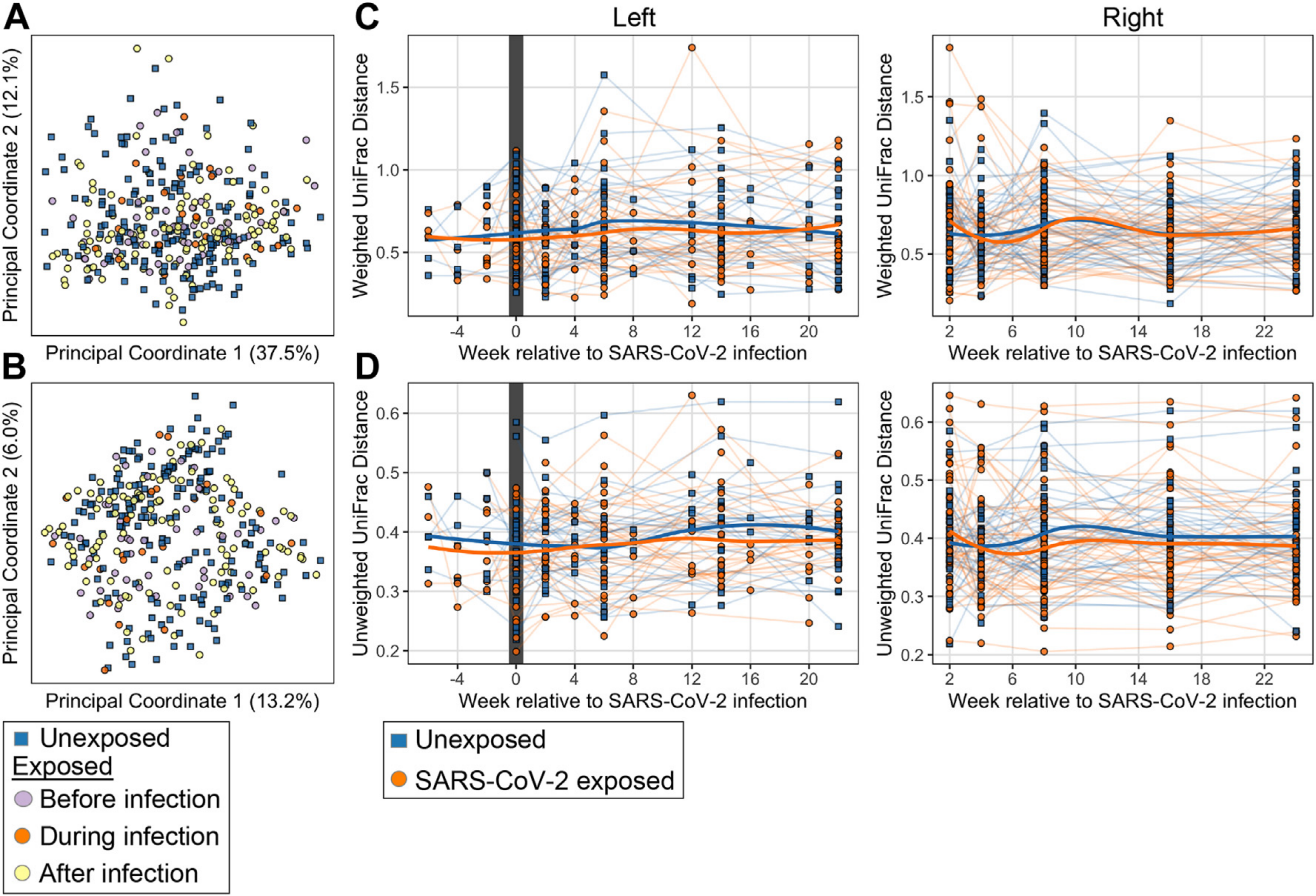
**Beta diversity of the salivary microbiome over time. Panels: A.** PCoA plots of unweighted and **B.** weighted UniFrac analyses of all timepoints for the 34 exposed subjects from before, during, and after viral positivity [colored by status relative to SARS-CoV2 infection] and their 31 matched unexposed subjects. **C.** Resilience of microbiome composition assessed by within-subject pairwise unweighted and **D**. weighted UniFrac distances between the first sample and later time points. **Left panels:** SARS-CoV-2-infected subjects (n = 34) before, during, and after viral positivity and their 31 matched unexposed subjects, with the study week shown relative to COVID infection with the week of the first positive test defined as week 0. **Right panels:** SARs-CoV-2-infected subjects (n = 47) who were virus-positive at the first study visit and their 41 matched unexposed subjects. No significant differences were found between exposed and unexposed subjects (p > 0.05) when controlling for age and week of sampling, using linear mixed effects modeling.

Next, we asked whether there were global effects of SARS-CoV-2 infection on the community composition of the salivary microbiota. We examined beta diversity in exposed participants with timepoints before, during, and after infection and their matched unexposed counterparts (BDA sub-cohort). We found no significant relationship in community composition relative to SARS-CoV-2 infection status using a PERMANOVA test comparing the beta diversity between the exposed group before, during, and after infection and the unexposed group as four categorial variables (Fig. 4A and B; PERMANOVA, p > 0.05). To control for variation in the baseline microbiome for each participant and to understand how microbiome community composition changed over time, we calculated the beta-diversity resilience which we assessed by within-participant pairwise unweighted and weighted UniFrac distances between the first sample and later time points (Fig. 4C and D). As anticipated, the unexposed participants had a non-significant amount of variation resilience over time (p > 0.05, linear mixed effects model). The SARS-CoV-2-infected cases exhibited a similar pattern of non-significant variation over time (p > 0.05, linear mixed effects model). Using a linear mixed effects model accounting for age, we also did not observe any significant differences between the infected or matched control participants at any time point (p > 0.05). Similar results were observed with metrics of beta-diversity not involving assumptions about phylogeny (Bray Curtis and Jaccard, data not shown). In total, these data indicate substantial resilience of the salivary microbiome both in SARS-CoV-2 unexposed people and in relation to SARS-CoV-2 infection.

### Effect of SARS-CoV-2 infection on the taxonomy of the salivary microbiome

**Fig. 5:**
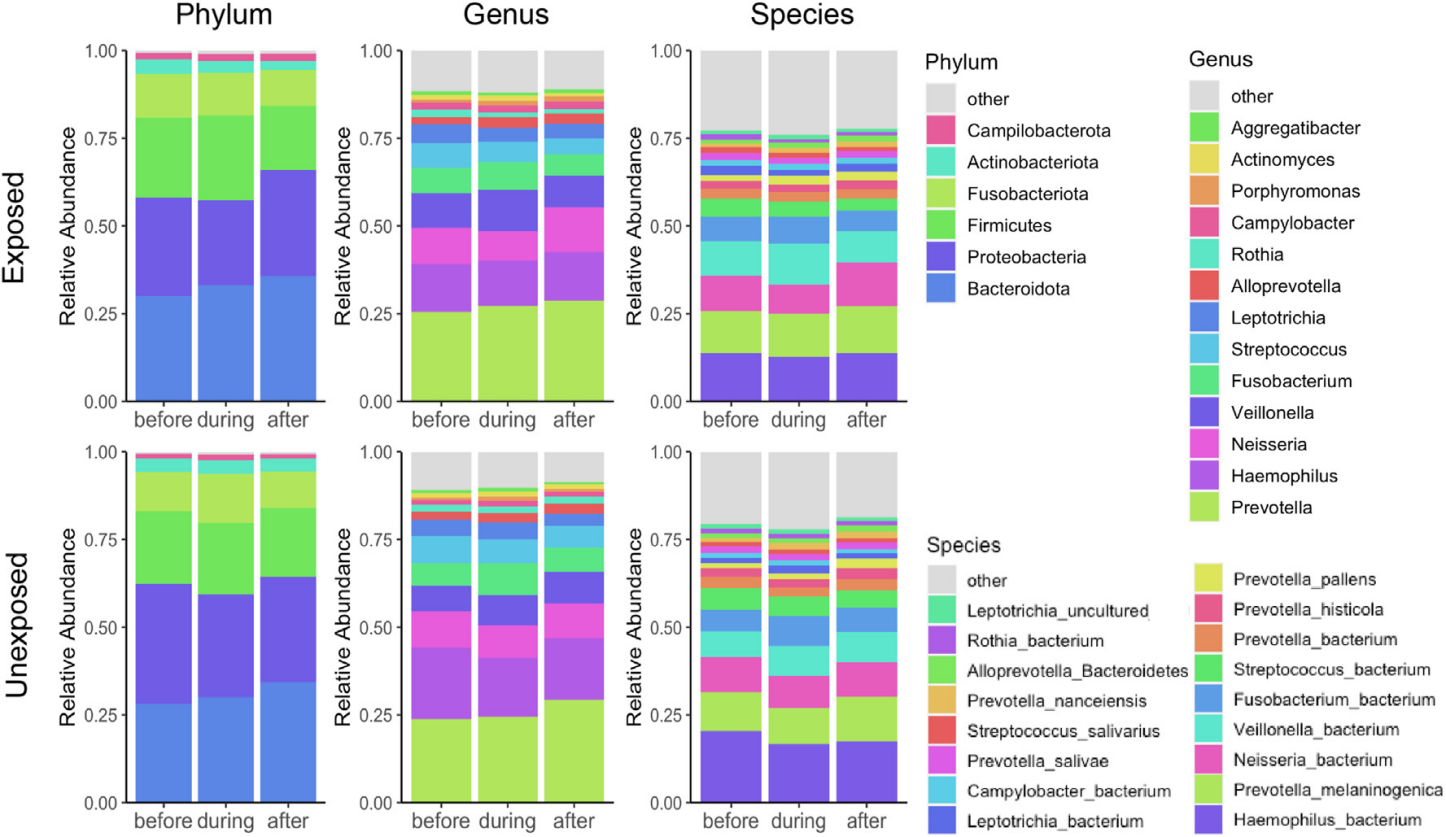
**Bar charts of taxon abundances in the salivary microbiome of subjects before, during, and after SARS-CoV-2 infection.** Mean relative abundance of taxa at the phylum, genus, and species level of 34 exposed subjects before, during, and after viral positivity and their 31 matched unexposed subjects using one sample per subject for each period in relation to infection. Taxa with mean relative abundance across all samples <1.5% are binned into ‘Other’. No significant differences were found between exposed and unexposed subjects (p > 0.05).

Next, we assessed whether any particular taxa were substantially affected by SARS-CoV-2 infection. We examined taxonomic differences at the phylum, genus, and species level in the SARS-CoV-2 infected cases and negative controls (Fig. 5) by examining the changes in abundances (delta abundances) within individuals between key timepoints.

To assess taxonomic changes that occurred during infection relative to before, we compared the change in abundance of the first SARS-CoV-2-negative timepoint before infection and the first SARS-CoV-2-positive timepoint within each individual. There were no significant differences in the delta abundance between SARS-CoV-2 exposed and unexposed at the phylum, genus, or species level (Student's T-test; FDR-corrected p > 0.05). Next, to assess taxonomic changes after recovery, we examined differences in the delta abundances between the first SARS-CoV-2-positive sample and a timepoint ≥8 weeks after infection. We found no significant differences in the change in taxa abundances between the SARS-CoV-2 exposed and unexposed individuals (Student's T test; FDR-corrected p > 0.05). Lastly, to examine the changes in the recovery samples relative to the baseline SARS-CoV-2-negative samples, we performed this same analysis comparing the delta abundance of the first SARS-CoV-2-negative timepoint before infection and a late recovered timepoint ≥8 weeks after infection in relation to the matched controls. No significant differences in these delta abundances were found between the SARS-CoV-2 exposed and unexposed groups (Student's T-test; FDR-corrected p > 0.05). Thus, in total, no specific taxonomic signal of change in abundance of phyla, genera, or species within individuals was associated with SARS-CoV-2 infection or recovery.

### Relationship of microbiome features with symptom severity

Next, we sought to elucidate relationships with symptom severity focusing only on the infected participants. To this end, we examined 4 timepoints for saliva samples from each of the.

81 SARS-CoV-2 exposed individuals as available: (i) before infection (median ± IQR: 14 ± 14 days before infection), (ii) the first positive sample during infection (defined as time_0_) (iii) early after infection as the first SARS-CoV-2-negative sample (14 ± 14 days after infection onset), and (iv) late after infection as the last sample obtained for that participant (154 ± 7 days after infection onset).

First, to address the impact of severity of the initial symptoms, we compared the microbiome from participants in three categories: asymptomatic or mildly symptomatic illness (n = 35), moderately symptomatic illness (n = 22), and severely symptomatic illness (n = 22), based on self-reported severity of symptoms (Table S2). There were significant differences in alpha diversity measures between the symptom severity groups only early after the illness. Those with severe illness had significantly reduced alpha diversity in the early after specimen compared to those who had asymptomatic or mildly symptomatic infection (Pielou evenness and observed ASVs, FDR-corrected p < 0.05, Kruskall–Wallis test with Dunn's post hoc test) and in those with moderately symptomatic infection (Pielou evenness, FDR-corrected p < 0.01) (Fig. S3A).

**Fig. 6:**
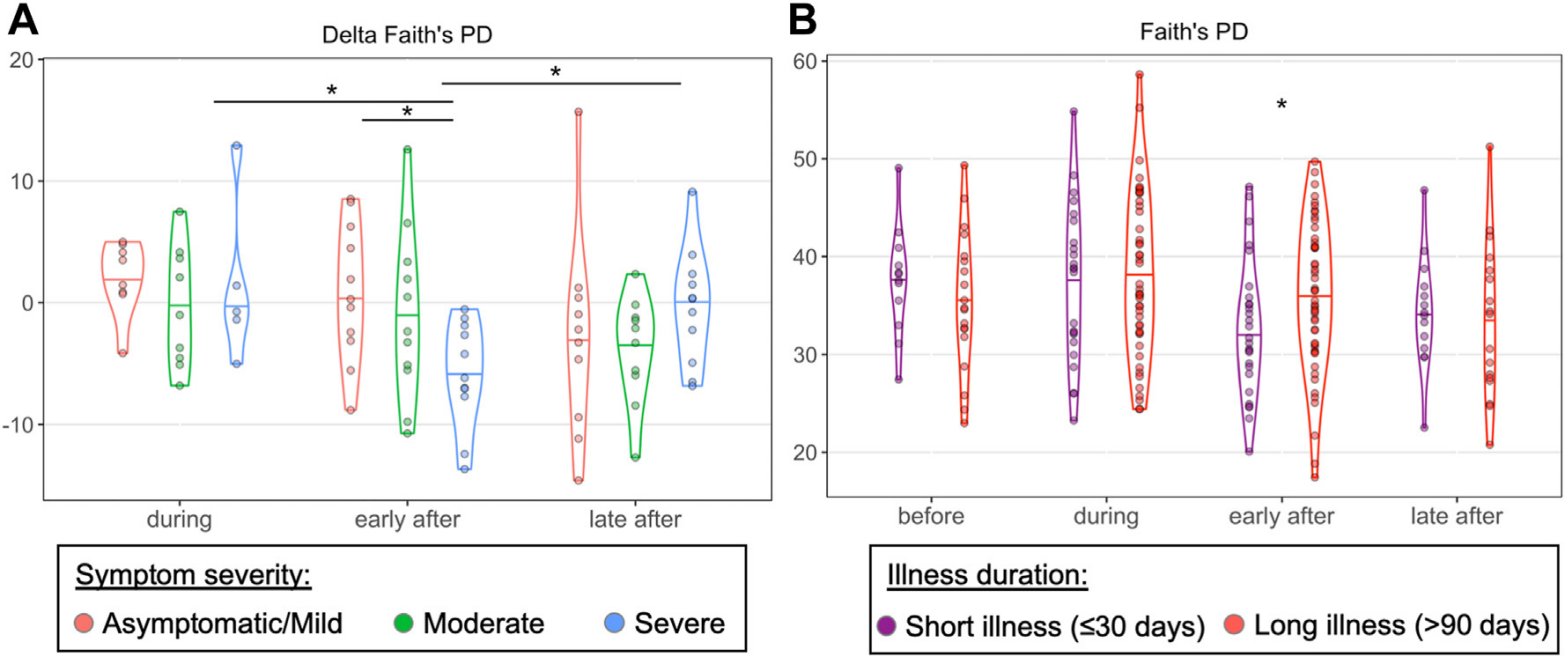
**Alpha diversity analysis in symptom severity and illness duration. A.** Change in alpha diversity as measured by Faith's phylogenetic diversity of the salivary microbiome comparing the values before SARS-CoV-2 infection in 81 subjects with those obtained during and after infection, according to the severity of their symptoms. Kruskal–Wallis test with Dunn's post hoc test FDR-correct p-values: ∗p < 0.05 **B.** Alpha diversity as measured by Faith's PD of the salivary microbiome of 79 SARS-CoV-2 infected subjects in samples obtained before, during, and after infection, according to the duration of the clinical illness. Mann–Whitney U test p-values: ∗p < 0.05. Before samples were obtained 14 ± 14 days before infection, early after were 14 ± 14 days after infection, and late after 154 ± 7 days after infection.

To control for individual variation of the microbiome at baseline, we also examined the relative change in alpha diversity during and after infection compared to the before values within exposed individuals (delta alpha diversity) (Fig. 6A, Fig. S3B). We found that in those with severe illnesses, the alpha diversity, as measured by Faith's PD, fell significantly in the early after specimens in relation to the baseline, which then recovered in the late after specimens (FDR-corrected p < 0.05, Kruskall–Wallis test with Dunn's post hoc test, Fig. 6A). The delta from baseline at the early after timepoint in those with severe infections also was significantly lower than observed in those with asymptomatic or mild illness (p < 0.05, Fig. 6A). Results from analyses of Observed Features and the Pielou metric showed similar, but not always significant differences between the deltas for the early after specimens in those with severe illnesses (Fig. S3B). In total, these data provide evidence in those with severe illnesses of a transient individual-specific decrease in alpha diversity of the salivary microbiome after infection that recovered within several months.

In our examination of beta-diversity, there were no apparent clustering according to severity related to the early after samples (Fig. S4A). However, at baseline (pre-infection), the samples from the participants with moderate illness were significantly more homogenous (less inter-participant distance) compared to the other groups in both UniFrac analyses; in the Weighted UniFrac analysis this difference persisted over the study course (Fig. S4B, FDR-corrected p < 0.01, Kruskall–Wallis test with Dunn's post hoc test). We interpret this as a founder difference between the clinical groups, and not related to SARS-CoV-2 infection. There were no significant differences for beta diversity within individuals over these timepoints (Fig. S4C, p > 0.05). Finally, we asked whether there were any significant differences in the taxa at the phylum, genus, or species level related to symptom severity at any of the timepoints or whether there were significant changes (delta) in abundance in individual participants over time according to illness severity. No significant differences were observed (linear mixed effects modeling p > 0.05 and Kruskall–Wallis test with Dunn's post hoc test FDR-corrected p > 0.05 respectively; data not shown).

### Relationship of microbiome features with duration of illness

To address effects of illness duration, we divided the SARS-CoV-2-positive cases into two groups: short illness duration [reported illness ≤30 days (n = 52, 64% of cases)] and long illness [reported illness >90 days (n = 27, 33% of cases)] (Fig. S1, Table S3). Only two cases, who were excluded from these analyses, did not fall in these distinct groups. This clear dichotomy allowed us to address whether there were differences in the salivary microbiome during the infection that could be related to illness duration. We addressed this question using the same before, during, early after, and late after samples relative to infection as above. Again, the most significant differences were related to the samples obtained from the early after infection period. At that timepoint, we found reduced alpha diversity measures in those with the shorter illness durations compared to those with the longer illnesses (Fig. 6B, Fig. S3C; p < 0.05, Mann–Whitney U test), which did not persist late after infection (p > 0.05, Mann–Whitney U test). We also examined individual-specific changes in alpha diversity by calculating the change between timepoints during and after infection relative to before within the same individual (delta alpha diversity). There were no significant differences in the delta alpha diversity according to illness duration group (Fig. S3D; p > 0.05, Mann–Whitney U test).

For beta diversity, there also were no significant differences between the groups at any time point, or in the individual-specific changes over time (Fig. S5; p > 0.05, Mann–Whitney U test**)**. However, the short illness duration group had significantly less distance (more homogeneity) within microbiome compositions during infection compared to those with a long illness duration, but greater distances before and after infection (Fig. S5; p < 0.05, Mann–Whitney U test). In analyses of taxa at the phylum, genus, and species levels, we did not find any significant changes between the groups differing in illness duration or in the change (delta) in abundance relative to baseline at any timepoint (linear mixed effects modeling p > 0.05 and Mann–Whitney U test p > 0.05 respectively; data not shown).

### Shotgun metagenomic analysis

We performed shotgun metagenomic sequencing on a sub-cohort of 62 subjects, selecting the 32 SARS-CoV-2 exposed individuals who had available samples before, during, and after infection and 30 matched unexposed counterparts (Table S4). This analysis focused on three timepoints with a single sample for each participant for each timepoint. The time points were the first study sample (as the Before), the first positive sample (During), and a sample from ≥8 weeks after the first positive sample (After) from the exposed group and samples from the corresponding study weeks for the matched unexposed individuals. While technical limitations prevented sequencing all timepoints for these participants, deep sequence data were available from 123 samples, including 51 samples from 17 participants at all three timepoints (Table S5). Samples were sequenced at a median depth of 18.3 ± 5.2 (IQR) GB per sample with a per sample median of 4.1 ± 5.6 GB mapping to microbial reads (Fig. S6). There were no significant differences in sequencing or microbial read depth or in percent of total reads that mapped to bacteria between cases and controls (Fig. S6; Mann–Whitney U test, p > 0.05).

**Fig. 7:**
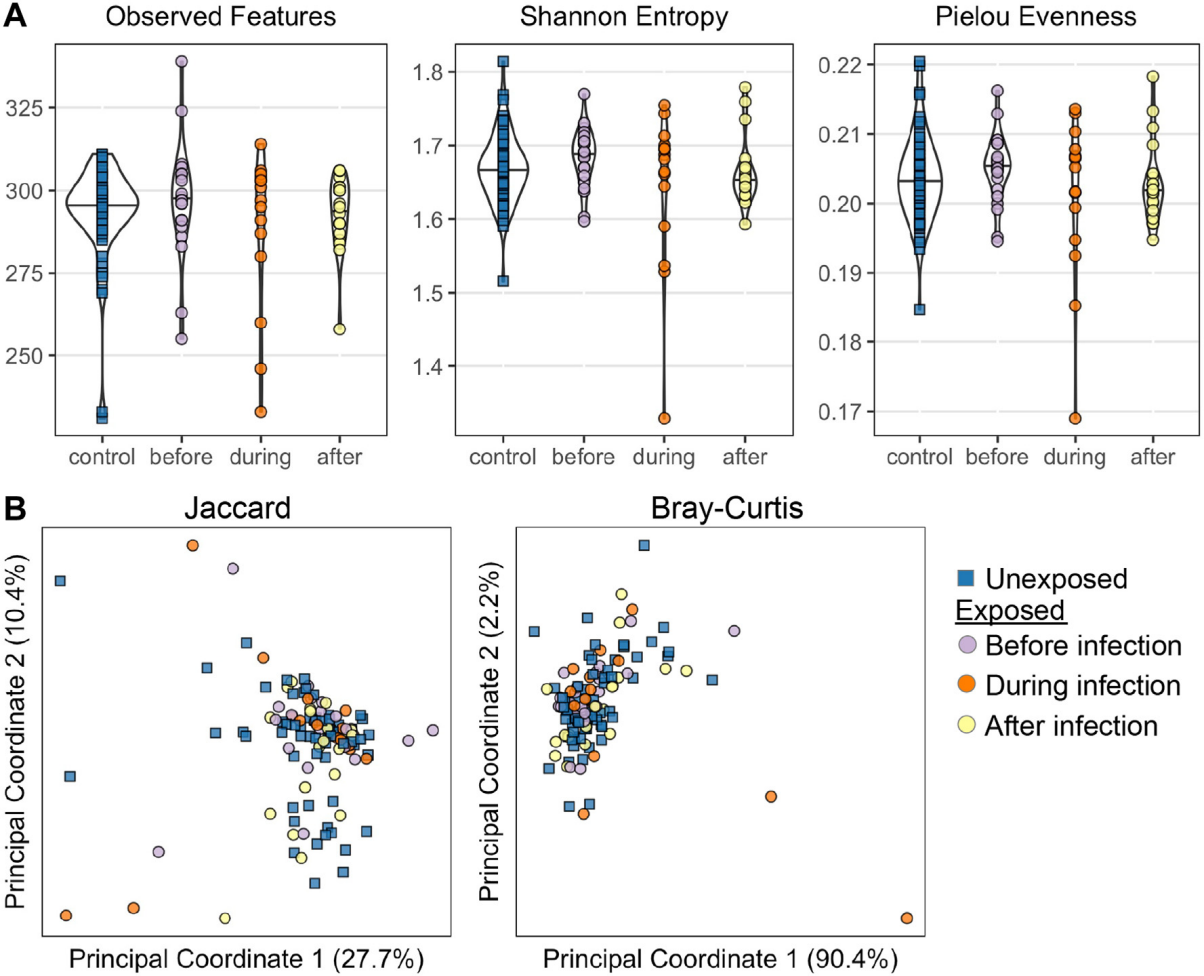
**Diversity analyses of functional gene pathways from metagenomics. A)** Alpha diversity metrics **B)** Beta diversity metrics.

To understand alteration in global microbial function during SARS-CoV-2 infection, we examined the difference of diversity and composition of functional gene pathways identified by HuMAnN3 between all SARS-CoV-2-infected cases and controls using both alpha (Fig. 7A) and beta (Fig. 7B) diversity metrics. There was no significant difference in alpha diversity metrics across timepoints in the SARS-CoV-2-infected cases or compared to controls (Fig. 7A, Kruskal–Wallis test, p > 0.05). Next, we examined the relationship between beta diversity and timepoint relative to infection or exposure status in the Jaccard or Bray–Curtis metrics of beta-diversity. Using a PERMANOVA test, we found that the beta diversity did not significantly differ between the exposed group before, during, and after infection and the unexposed group (Fig. 7B, PERMANOVA test, p > 0.05).

To examine specific functional gene pathways that may change in response to SARS-CoV-2 infection, we considered three main relationships: (1)whether there was significant variation over time in control samples as a measure of natural variation, (2)differences in pathway abundances between controls and before infection samples in the SARS-CoV-2-infected cases, and (3) how functional pathways might vary during SARS-CoV-2 infection relative to before and after. To reduce the number of comparisons generated by such analyses, we used a co-occurrence, guild-based approach in which highly co-occurring pathways are binned together, using SCNIC.^46^ In short, groups of highly co-occurring functional pathways were binned together and analyzed as single units (Fig. S7A, Table S6). We used linear mixed effects modeling implemented using MaAslin2^43^ to address the three questions posed above, but did not find any significant differences in feature abundance in any analysis (FDR-corrected p-value >0.05). Visualizing the data showed no significant clustering of samples or features (Fig. S7B).

## Discussion

In this work, we examined the microbiome in expectorated saliva of individuals with and without SARS-CoV-2 infection, in contrast to previous reports of analyses of the gut,^15^, ^16^, ^17^, ^18^, ^19^, ^20^, ^21^, ^22^ nasopharyngeal,^47^, ^48^, ^49^, ^50^, ^51^, ^52^, ^53^ and/or oropharyngeal^47^^,^^52^, ^53^, ^54^, ^55^, ^56^, ^57^, ^58^ microbiomes in SARS-CoV-2 infection. We chose expectorated saliva because many prior (non-COVID-19) studies of the oral microbiome have used this approach^59^, ^60^, ^61^, ^62^, ^63^ and it can be a proxy for the lung and respiratory tract microbiome.^25^, ^26^, ^27^ We prospectively obtained saliva specimens routinely across the entire 24-week study period that had been used to detect SARS-CoV-2 infection through qPCR.^23^^,^^24^ To our knowledge, we report the first community-based study that examines the effects of SARS-CoV-2 infection on the salivary microbiome. Since viral detection from saliva samples is less invasive than nasopharyngeal or oropharyngeal collection, its on-going use for detecting SARS-CoV-2 infection may provide numerous samples for future host-microbiome analyses.

Another distinctive study feature is that all of the samples were obtained in early 2020, in the initial COVID-19 wave in the United States. The infections were exclusively due to the Wuhan-Hu-1 SARS-CoV-2 strain, and no participant had yet received SARS-CoV-2 vaccination. This study, conducted in an immunologically naïve population, avoids the confounding effects of different circulating virus variants, and of heterogeneous SARS-CoV-2 immune status, and allows us to study the dynamics of the salivary microbiome before, during, and after SARS-CoV-2 infection. Although infection with such an early strain of the virus may not be fully relevant to current populations and infections, these findings provide a unique view of initial SARS-CoV-2 related changes in the oral microbiome. They also provide baseline knowledge for studies on subsequent infections and/or on cases of long COVID. Prior studies have suggested long-term impact of SARS-CoV-2 infection on the intestinal microbiome bacteria and bacteriophages due to SARS-CoV-2 infection,^64^, ^65^, ^66^ issues that should be evaluated in future studies.

A third distinguishing feature of this study is that most of the SARS-CoV-2 infections were generally mild and required minimal intervention, reflecting the bulk of community infections worldwide.^67^ This contrasts with other microbiome studies in COVID-19 patients that largely focused on those hospitalized^22^^,^^47^^,^^48^^,^^52^^,^^53^^,^^55^, ^56^, ^57^, ^58^^,^^68^, ^69^, ^70^; such patients usually had much more severe infections and often received multiple therapies including antibiotics prior to or throughout microbiota ascertainment. As a result, our findings may paint a more accurate and relevant picture of the effects of SARS-CoV-2 infection on the upper respiratory track microbiome in most cases and in the absence of significant treatment.

However in terms of generalizability, it is important to note that our study population represents a specific subset of individuals, primarily consisting of healthcare workers, during a particular period in time who may have had distinct exposures and protective measures compared to the general public during the early stages of the COVID-19 pandemic. We emphasize that our study did not specifically analyze or compare the outcomes between healthcare workers (HCWs) and non-HCWs, nor did we directly compare our cohort's characteristics with local contemporaneous community data. However, while our findings may not be directly generalizable to the broader population, they provide important information for understanding the microbiome dynamics in individuals with occupational exposure to SARS-CoV-2.

Additionally, while the severity of COVID-19 disease can vary between males and females,^71^ this study did not aim to investigate the sex-specific differences. The distribution of infected participants in our study reflects the natural infection rate of both men and women in the investigated population, and we used sex as a matching criterion when selecting unexposed participants. Our primary focus was on examining the changes in the salivary microbiome in relation to SARS-CoV-2 infection. However, considering sex as a potential factor, particularly in investigations of disease severity, in future studies could contribute to a more comprehensive understanding of the disease and its impact on different populations.

Our findings indicate that the oral microbiome is resilient to perturbation by mild to moderate SARS-CoV-2 infection as evidenced by the lack of substantial change in its ecological and taxonomic characteristics. These findings of bacterial stability during infection also are consistent with the infrequency of overt bacterial pneumonia in the acute aftermath of mild SARS-CoV-2 infections.^67^^,^^72^ While we observed stochastic changes within individual participants, the alpha and beta diversity across all participants remained relatively consistent over time and not impacted by mild or moderate SARS-CoV-2 infection. In contrast, other studies that show changes in the gut and respiratory microbiome in mild to moderate SARS-CoV-2 infection may reflect concomitant medical treatments, especially antibiotic use,^22^^,^^55^ as our recent report also indicated^15^; however, reporting on treatments within these cohorts has been inconsistent. Although studies of hospitalized patients with influenza^54^ or other acute respiratory illness^47^ indicate uniqueness of respiratory track microbial signatures for SARS-CoV-2 infection, it remains unclear whether these reflect the infection or the treatments administered.

Most reports showing significant upper respiratory track microbiome changes in SARS-CoV-2 infection focused on patients hospitalized and/or with severe symptoms.^47^^,^^52^^,^^53^^,^^58^ Although most participants in our cohort were those with mild or moderate symptoms, among those with severe symptoms, microbiome alpha diversity was significantly reduced compared to those less ill, an effect peaking early after infection and returning to baseline in later samples. Although there was little antibiotic use overall in this cohort, it was significantly greater in those with severe symptoms (Table S2), which may be driving these findings. Despite the significant diversity changes in those with severe symptoms, no conserved compositional or taxonomic changes were identified even when accounting for individual variation at baseline. The lack of significance may reflect relatively low subject numbers in the individual groups, or that alterations of specific oral taxa in severe SARS-CoV-2 infection are highly individual specific. Studies, including the present, that report the strongest respiratory track microbiome changes in hospitalized patients with severe infection,^47^^,^^52^^,^^53^^,^^58^ suggest that major exogenous factors including treatments or severe immune dysfunction are necessary to perturb this resilient microbial niche. Nevertheless, substantial changes in the respiratory track microbiome in hospitalized patients are not universal.^48^^,^^58^^,^^70^

We observed that SARS-CoV-2-exposed individuals with shorter illness duration (≤30 days) have significantly greater microbiome compositional homogeneity during infection compared to those with long illness, despite less homogeneity before and after infection, which suggests the existence of a conserved, acute responses to the infection. Significantly lower alpha diversity early after infection in those with short illness duration also suggests a potentially adaptive microbiome compositional response to infection that may aid in earlier recovery. Although we were not able to identify specific microbial taxa conserved within this group, further microbiome studies in relation to illness duration are potentially relevant to the development of late complications.

To our knowledge, no other studies have examined serial microbial samples from individuals before, during, and after SARS-CoV-2 infection. As such, differences observed in prior studies not associated with disease severity or antibiotic treatment may be attributed to the substantial interpersonal variation in the upper respiratory track microbiome. In contrast, our study directly examines the temporal changes in microbiome communities within individuals as well as across SARS-CoV-2-exposed and unexposed groups. We found that mild to moderate SARS-CoV-2 infection does not lead to significant alterations in the salivary microbiome beyond the natural stochasticity observed over time in non-infected people. However, we did find a significant reduction in diversity early after infection (14 ± 14, median ± IQR, days after infection) in SARS-CoV-2-exposed individuals with severe illness compared to the diversity of their saliva microbiome before infection. These findings highlight the relevance of our work in understanding the stability and adaptability of the salivary microbiome in the context of SARS-CoV-2 infection and the potential importance of greater monitoring of those with severe symptoms. Understanding the dynamic interplay between host and microbiome during viral infections may aid in developing strategies to potentially mitigate the impact of those infections.

In conclusion, we observed relative stability of the oral microbiome over the course of SARS-CoV-2 infection, with minimal impact from mild to moderate infection, but with significant early changes in those with severe illness, as well as more conserved changes in those with short (≤30 days) illness duration. These findings suggest symptom severity and duration as important factors related to oral microbiome alterations. As a biological niche implicated in oral,^73^^,^^74^ pulmonary,^26^^,^^75^ and systemic^73^ health, the salivary microbiome is an interface worthy of further exploration in SARS-CoV-2 patients.

## Contributors

Obtained and analyzed microbiome data: AJSA**,** obtained clinical data: DBH, ESB, TA, PG**,** analyzed clinical data: DBH, ESB, JR, MLG, TA, PG**,** enrolled patients: JLC, RP**,** constructed study cohort: DBH, ESB**,** project administration: MJB, funding acquisition: MJB**,** supervision: MJB**,** visualization: AJSA**,** writing – original draft: AJSA, MJB**,** writing – review & editing: AJSA, DBH, JT, MLG, JLC, RP, ESB, MJB, accessed and verified the data: AJSA, MJB, decision to submit the manuscript: AJSA, MJB.

## Data sharing statement

16S rRNA gene amplicon sequencing data is publicly available at EBI/ENA (https://www.ebi.ac.uk/ena) accession number PRJEB62655 and QIITA (https://qiita.ucsd.edu) study ID 15066. Shotgun metagenomic sequencing data is available at EBI/ENA accession number PRJEB62577. All analysis code is available upon reasonable request.

## Declaration of interests

The authors declare that there are no competing interests.
