# Supplementary material for: Saliva microbiome in relation to SARS-CoV-2 infection in a prospective cohort of healthy US adults
Source: eBioMedicine. 2023 Jul 22;94:104731. doi: 10.1016/j.ebiom.2023.104731 (PMC10382861; doi:10.1016/j.ebiom.2023.104731)
Supplement: Supplementary Figs and Tables [file mmc1.pdf]

Table S1. Clinical summary for the 138 subjects with 16S rRNA gene sequencing of saliva samples

	Full Cohort†		During After (DA) cohort		Before During After (BDA) cohort	
	**Unexposed**	**Exposed**	**Unexposed***	**Exposed**	**Unexposed***	**Exposed**
**Subjects**	57	81	41	47	31	34
**Total samples**	313	435	224	256	176	179
**Sex: females**	41 (71.9)	60 (74.1)	29 (70.7)	34 (72.3)	24 (77.4)	26 (76.5)
**Age**						
**20-39**	31 (54.4)	45 (55.6)	23 (56.1)	28 (59.6)	16 (51.6)	17 (50)
**40-59**	18 (31.6)	25 (30.9)	13 (31.7)	12 (25.5)	10 (32.3)	13 (38.2)
**over 60**	7 (12.3)	11 (13.6)	5 (12.2)	7 (14.9)	4 (12.9)	4 (11.8)
**Race**						
**White**	27 (47.4)	44 (54.3)	17 (41.5)	23 (48.9)	18 (58.1)	21 (61.8)
**Asian**	9 (15.8)	10 (12.3)	7 (17.1)	6 (12.8)	5 (16.1)	4 (11.8)
**Black**	9 (15.8)	13 (16)	8 (19.5)	9 (19.1)	4 (12.9)	4 (11.8)
**other**	9 (15.8)	12 (14.8)	7 (17.1)	8 (17)	3 (9.7)	4 (11.8)
**Ethnicity: Hispanic**	11 (19.3)	17 (21)	11 (26.8)	14 (29.8)	3 (9.7)	3 (8.8)
**Smoking**						
**Never**	39 (68.4)	51 (63)	30 (73.2)	29 (61.7)	20 (64.5)	22 (64.7)
**Past**	14 (24.6)	27 (33.3)	9 (22)	15 (31.9)	9 (29)	12 (35.3)
**Current**	3 (5.3)	3 (3.7)	2 (4.9)	3 (6.4)	1 (3.2)	0 (0)
**Comobidities**						
**Any**	30 (52.6)	43 (53.1)	23 (56.1)	26 (55.3)	16 (51.6)	17 (50)
**Diabetes**	5 (8.8)	2 (2.5)	5 (12.2)	2 (4.3)	0 (0)	0 (0)
**Hypertension**	9 (15.8)	17 (21)	6 (14.6)	10 (21.3)	3 (9.7)	7 (20.6)
**Cardiovascular disease**	1 (1.8)	3 (3.7)	1 (2.4)	3 (6.4)	0 (0)	0 (0)
**Asthma**	9 (15.8)	10 (12.3)	6 (14.6)	6 (12.8)	6 (19.4)	4 (11.8)
**Autoimmune disease**	4 (7)	4 (4.9)	2 (4.9)	1 (2.1)	3 (9.7)	3 (8.8)
**Symptoms**						
**Asymptomatic**		9 (11.1)		7 (14.9)		2 (5.9)
**Mild**		26 (32.1)		18 (38.3)		8 (23.5)
**Moderate**		23 (28.4)		10 (21.3)		13 (38.2)
**Severe**		23 (28.4)		12 (25.5)		11 (32.4)
**Symptom duration**						
**< 10 days**		28 (34.6)		19 (40.4)		9 (26.5)
**10-29 days**		25 (30.9)		11 (23.4)		14 (41.4)
**30+ days**		24 (29.6)		14 (29.8)		10 (29.4)
**missing**		4 (4.9)		3 (6.4)		1 (2.9)
**Days ill; median +/- IQR**		17 +/- 121		14 +/- 126		29 +/- 113
**Treatments**						
**ER**		7 (8.6)		2 (4.3)		5 (14.7)
**Hospital admission**		3 (3.7)		1 (2.1)		2 (5.9)
**Antibiotics**		5 (6.1)		2 (4.3)		3 (8.8)
**Corticosteroid**		1 (1.2)		1 (2.1)		0 (0)
**Anticoagulant**		2 (2.5)		1 (2.1)		1 (2.9)
**Hydroxychloroquine**		6 (7.4)		2 (4.2)		4 (11.8)
**Remdesivir**		1 (1.2)		0 (0)		1 (2.9)

Values reported as n (%) unless otherwise stated
†Includes all samples in the DA and BDA cohorts
*Subset of unexposed are included in both DA and BDA cohorts based on matching criteria with exposed

Table S2. Clinical characteristics of 79 SARS-CoV-2 infected subjects, according to symptom severity

	Aymptomatic/ Mild	Moderate	Severe	p-value*
**n**	35	22	22	
**sex (females)**	27 (77.1)	18 (81.8)	14 (63.6)	
**Age**				0.08
**20-39**	21 (60)	15 (68.2)	8 (36.4)	
**40-59**	8 (22.9)	4 (18.2)	12 (54.5)	
**60+**	6 (17.1)	3 (13.6)	2 (9.1)	
**Race**				0.43
**Asian**	6 (17.1)	0 (0)	3 (13.6)	
**Black**	4 (11.4)	5 (22.7)	3 (13.6)	
**White**	20 (57.1)	13 (59.1)	11 (50)	
**Other**	5 (14.3)	4 (18.2)	3 (13.6)	
**Hispanic**	5 (14.3)	7 (31.8)	5 (22.7)	0.3
**Smoking**				0.81
**Never**	20 (57.1)	15 (68.2)	15 (68.2)	
**Past**	13 (37.1)	6 (27.3)	7 (31.8)	
**Current**	2 (5.7)	1 (4.5)	0 (0)	
**Co-mobidities**				
**Any**	19 (54.3)	8 (36.4)	15 (68.2)	0.12
**Diabetes**	2 (5.7)	0 (0)	0 (0)	0.5
**Hypertension**	6 (17.1)	4 (18.2)	7 (31.8)	0.3
**Cardiovascular disease**	2 (5.7)	1 (4.5)	0 (0)	0.79
**Asthma**	5 (14.3)	1 (4.5)	4 (18.2)	0.39
**Autoimmune disease**	2 (5.7)	0 (0)	2 (9.1)	0.38
**Antibiotic use**	0 (0)	1 (4.5)	4 (18.2)	**0.01**
**Timepoint (day)****				0.76
**before**	11 (-14 ± 14)	12 (-14 ± 14)	10 (-14 ± 7)	
**during**	34 (0 ± 0)	20 (0 ± 0)	17 (0 ± 0)	
**early after**	34 (14 ± 10.5)	22 (28 ± 14)	22 (28 ± 14)	
**late after**	11 (140 ± 28)	12 (154 ± 14)	10 (154 ± 7)	

values reported as n (%) unless otherwise stated

*Fisher's exact test

**n = number of subjects (median sample day ± IQR)

Table S3. Clinical characteristics of 79 SARS-CoV-2 infected subjects, according to illness duration

	short illness (≤ 30 days)	long illness (> 90 days)	p-value*
**n**	52	27	
**sex (females)**	40 (76.9)	19 (70.4)	0.59
**Age**			0.62
**20-39**	31 (59.6)	13 (48.1)	
**40-59**	14 (26.9)	10 (37)	
**60+**	7 (13.5)	4 (14.8)	
**Race**			0.63
**Asian**	6 (11.5)	3 (11.1)	
**Black**	6 (11.5)	6 (22.2)	
**White**	31 (59.6)	13 (48.1)	
**Other**	9 (17.3)	5 (18.5)	
**Hispanic**	12 (23.1)	5 (18.5)	0.78
**Smoking**			0.83
**Never**	34 (65.4)	16 (59.3)	
**Past**	16 (30.8)	10 (37)	
**Current**	2 (3.8)	1 (3.7)	
**Comobidities**			
**Any**	23 (44.2)	19 (70.4)	0.34
**Diabetes**	2 (3.8)	0 (0)	0.54
**Hypertension**	9 (17.3)	8 (29.6)	0.14
**Cardiovascular disease**	2 (3.8)	1 (3.7)	1
**Asthma**	5 (9.6)	5 (18.5)	0.3
**Autoimmune disease**	1 (1.9)	3 (11.1)	0.11
**Symptoms**			**0.03**
**Asymptomatic**	9 (17.3)	0 (0)	
**Mild**	19 (36.5)	7 (25.9)	
**Moderate**	13 (25)	9 (33.3)	
**Severe**	11 (21.2)	11 (40.7)	
**antibiotic use**	4 (7.7)	1 (3.7)	0.66
**Timepoints****			
**before**	19 (-14 ± 14)	14 (-14 ± 10.5)	
**during**	48 (0 ± 0)	23 (0 ± 0)	
**early after**	51 (14 ± 14)	27 (28 ± 21)	
**late after**	19 (154 ± 14)	14 (154 ± 14)	

values reported as n (%) unless otherwise stated
*Fisher's exact test
**n (median sample day ± IQR)

Table S4. Clinical summary for the 62 subjects with shotgun metagenomics sequencing of saliva samples

	Full Cohort†		Before During After (BDA) cohort	
	Unexposed	Exposed	Unexposed	Exposed
Subjects	30	32	11	6
Total samples	64	59	33	18
Sex: females	24 (80)	24 (75)	8 (72.7)	3 (50)
Age				
20-39	15 (50)	16 (50)	2 (18.2)	3 (50)
40-59	11 (36.7)	12 (37.5)	6 (54.5)	2 (33.3)
over 60	4 (13.3)	4 (12.5)	3 (27.3)	1 (16.7)
Race				
White	19 (63.3)	19 (59.4)	8 (72.7)	6 (100)
Asian	4 (13.3)	4 (12.5)	1 (9.1)	0 (0)
Black	4 (13.3)	4 (12.5)	0 (0)	0 (0)
other	3 (10)	4 (12.5)	2 (18.2)	0 (0)
Ethnicity: Hispanic	3 (10)	3 (9.4)	1 (9.1)	0 (0)
Smoking				
Never	20 (66.7)	20 (62.5)	4 (36.4)	4 (66.7)
Past	9 (30)	12 (37.5)	7 (63.6)	2 (33.3)
Current	1 (3.3)	0 (0)	0 (0)	0 (0)
Comobidities				
Any	17 (56.7)	16 (50)	8 (72.7)	2 (33.3)
Diabetes	0 (0)	0 (0)	0 (0)	0 (0)
Hypertension	3 (10)	7 (21.9)	2 (18.2)	2 (33.3)
Cardiovascular disease	0 (0)	0 (0)	0 (0)	0 (0)
Asthma	6 (20)	4 (12.5)	2 (18.2)	0 (0)
Autoimmune disease	3 (10)	3 (9.4)	2 (18.2)	0 (0)
Symptoms				
Asymptomatic	0 (0)	2 (6.3)	0 (0)	0 (0)
Mild	0 (0)	8 (25)	0 (0)	3 (50)
Moderate	0 (0)	11 (34.4)	0 (0)	1 (16.7)
Severe	0 (0)	11 (34.4)	0 (0)	2 (33.3)
Symptom duration				
< 10 days		8 (25)		1 (16.7)
10-29 days		13 (40.6)		2 (33.3)
30+ days		10 (31.3)		3 (50)
missing		1 (3.1)		0 (0)
Days ill; median +/- IQR		29 +/- 111		79.5 +/- 117.25
Treatments				
ER		5 (15.6)		1 (16.7)
Hospital admission		2 (6.3)		0 (0)
Antibiotics		3 (9.4)		0 (0)

Values reported as n (%) unless otherwise stated
†Includes all samples in the BDA cohort

Table S5. Sample breakdown for the 123 samples for which shotgun metagenomics data were obtained

	Unexposed n=30	Exposed n=32	Total samples
All 3 time points	11	6	51
2 time points only	12	15	54
Before & during infection	8	4	24
Before & after infection	2	8	20
During & after infection	2	3	10
1 time point only	7	11	18
Before infection	3	6	9
During infection	1	2	3
After infection	3	3	6

Table S6. Co-occurrence guild membership

See separate excel spreadsheet containing the table

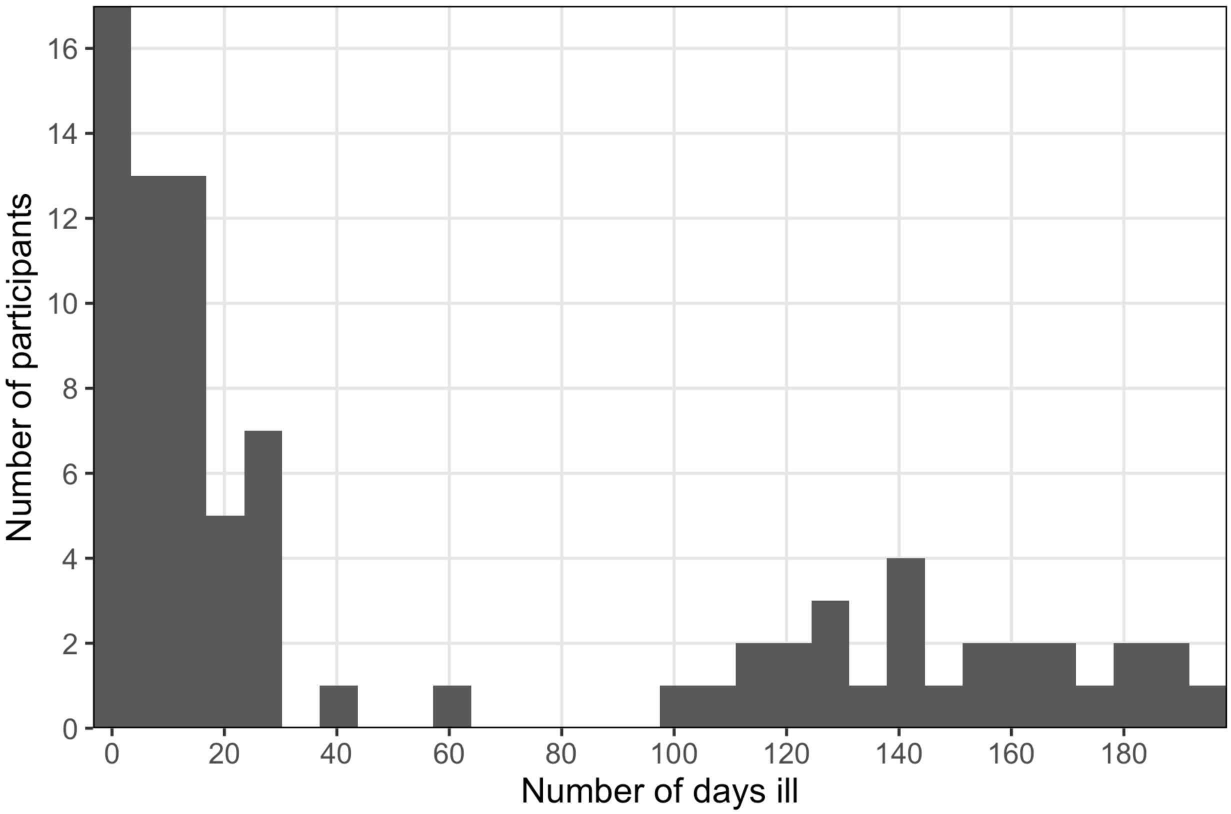

Figure S1. Histogram of the days ill for the 81 subjects infected with SARS-CoV-2.

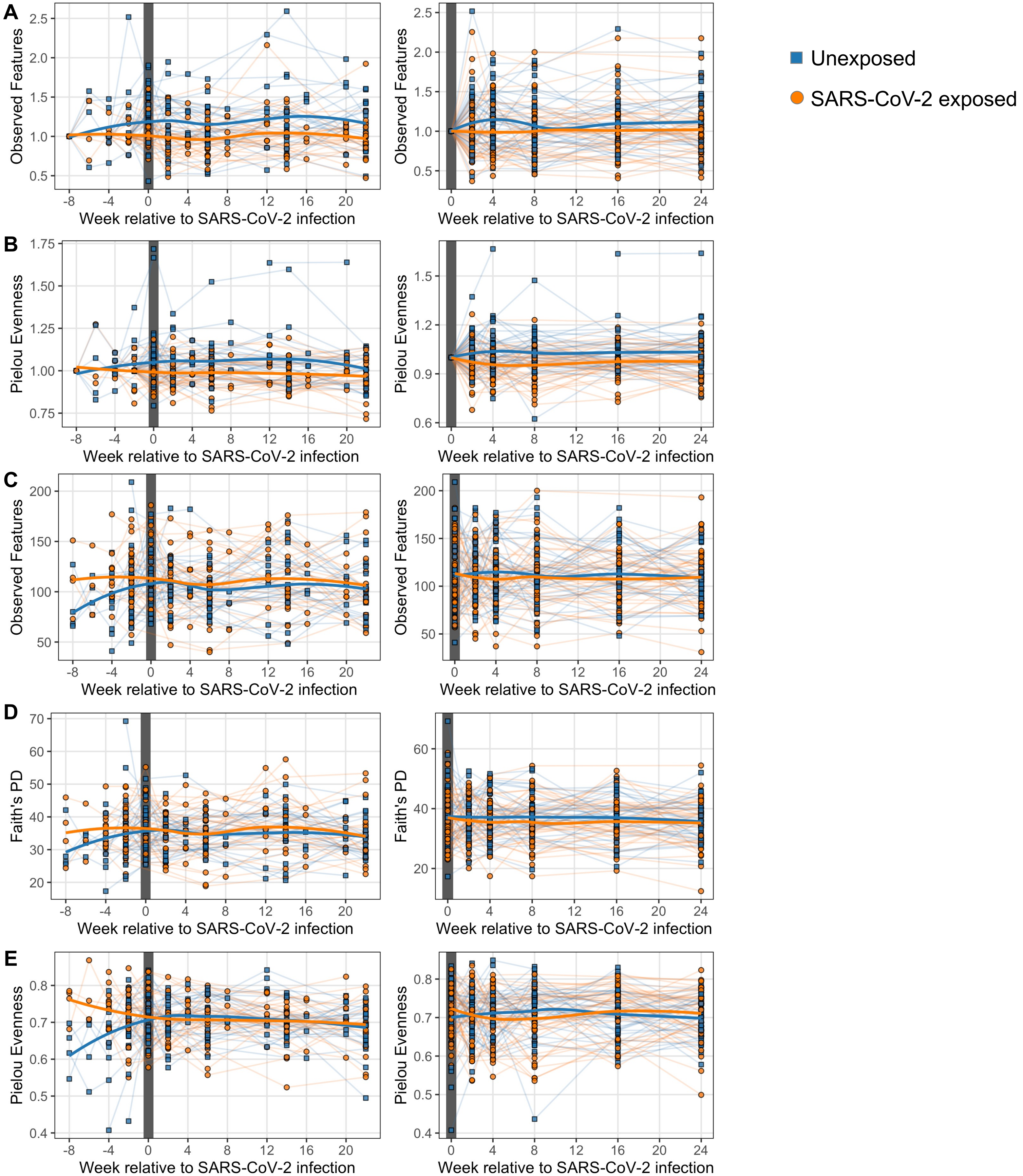

Figure S2. Alpha diversity over time of the salivary microbiome. Alpha diversity normalized based on proportion of the first timepoint for each individual reported as **A.** Observed features (ASVs) and **B.** Pielou Evenness values in proportion to the week zero sample and without normalization **C.** Observed features (ASVs) **D.** Faith's Phylogenetic Diversity (PD) and E. Pielou Evenness **Left Panels:** SARS-CoV-2 infected subjects (n=34) before, during, and after viral positivity and their 31 matched controls, with the study week shown relative to COVID infection with the week of the first positive test defined as week 0. **Right panels:** SARS-CoV-2-infected subjects (n=47) who were virus-positive at the first study visit and their 41 matched controls. There were no significant differences in either analysis between infected subjects and controls (p > 0.05) when controlling for age and week of sampling, using linear mixed effects modeling.

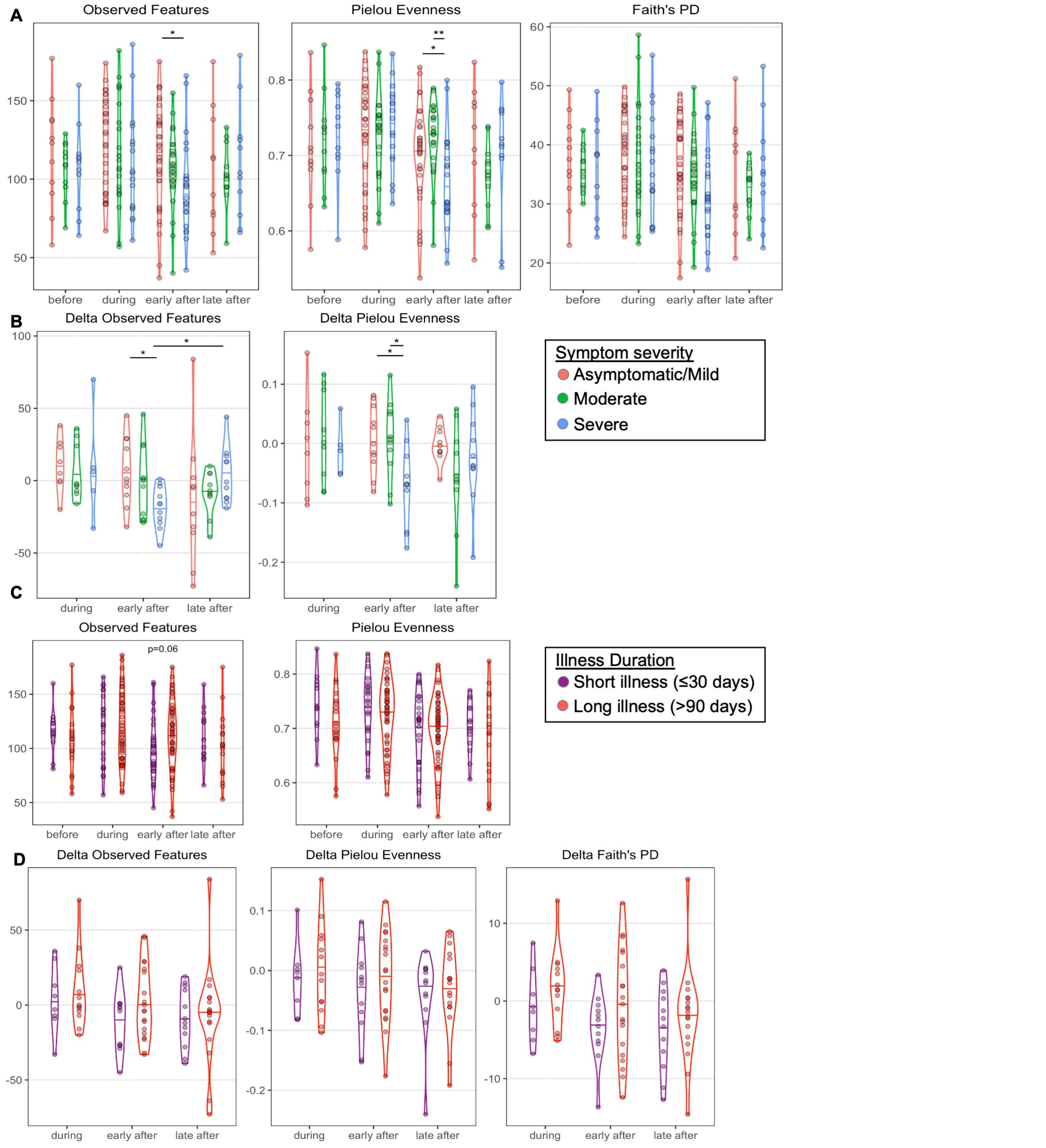

Figure S3. Alpha diversity of the salivary microbiome of SARS-CoV-2 infected persons throughout infection. A. Alpha diversity stratified by symptom severity determined using Observed features (ASVs)**,** Pielou Evenness, and Faith's Phylogenetic Diversity (PD) metrics. **B.** Change in alpha diversity stratified by symptom severity comparing the values before SARS-CoV-2 with those obtained during and after infection determined using Observed features and Pielou Evenness. **C.** Alpha diversity stratified by illness duration determined using Observed features (ASVs) and Pielou Evenness metrics. **D.** Change in alpha diversity stratified by illness duration of the salivary microbiome comparing the values before SARS-CoV-2 with those obtained during and after infection determined using Observed features and Pielou Evenness, and Faith's Phylogenetic Diversity (PD). Before samples were obtained obtained 14 ± 14 days before infection, early after were 14 ± 14 days after infection, and late after 154 ± 7 days after infection. Kruskall-Wallis test with Dunn's post hoc test FDR-correct p-values: * p < 0.05, ** p < 0.01

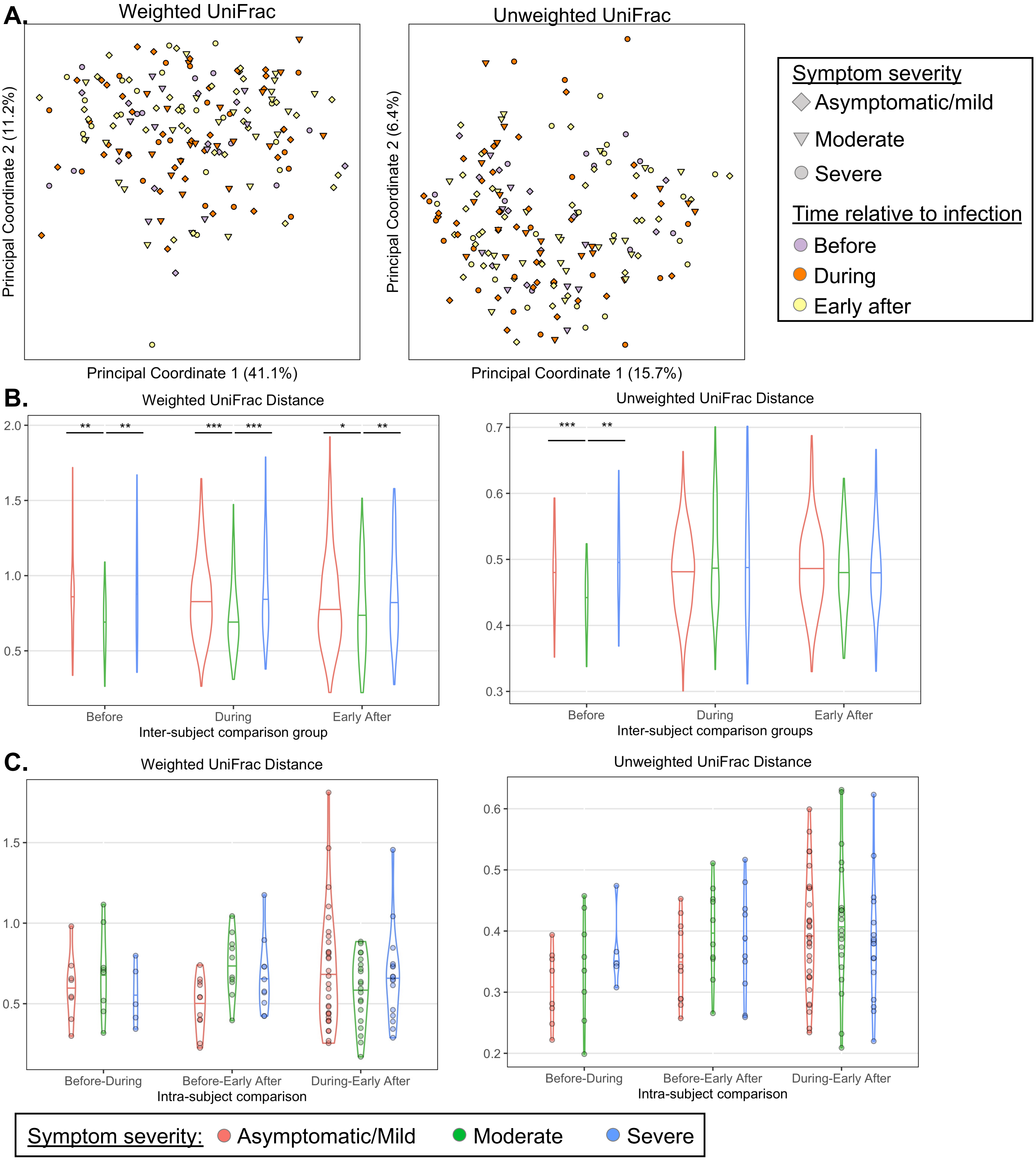

Figure S4. Beta diversity of the salivary microbiome in 81 SARS-CoV-2 subjects before, during, and early after infection, according to symptom severity. A. Weighted and unweighted UniFrac PCoA plots showing asymptomatic/mild subjects before (n=11), during (n=34), and early after (n=34) infection; moderate symptom subjects before (n=12), during (n=20), and early after (n=22) infection; and severe symptom subjects before (n=10), during (n=17), and early after (n=22) infection. **B.** Inter-subject weighted and unweighted UniFrac distances within symptom severity groups comparing within each severity group before, during, or early after infection. **C.** Intra-subject distances (distance between timepoints for individual subjects) broken up by the three severity groups. Kruskall-Wallis test with Dunn's post hoc test FDR-correct p-values: ** p < 0.01, *** p <0.001.

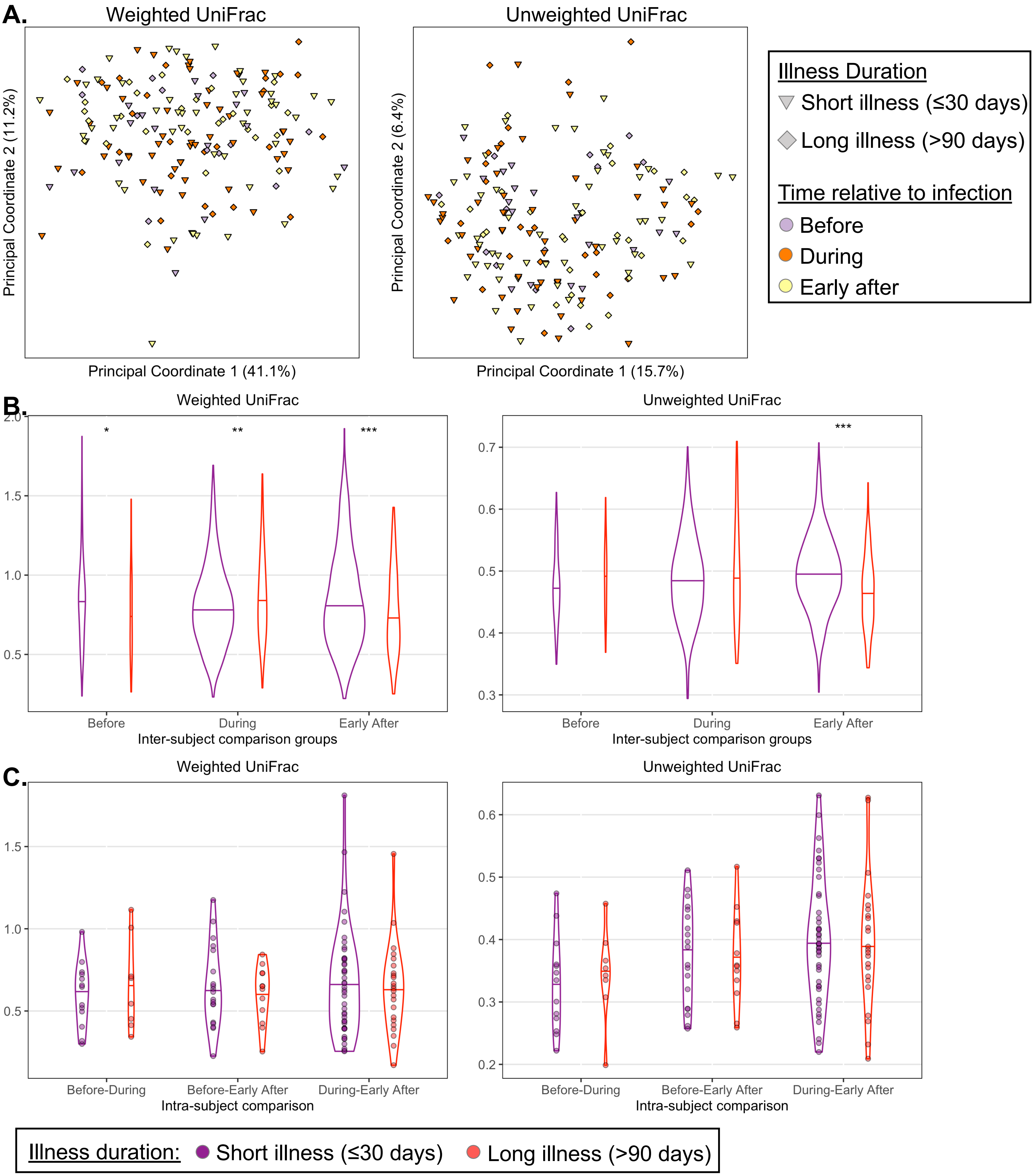

Figure S5. Beta diversity of SARS-CoV-2 cases by illness duration before, during, and early after infection. A. Weighted and unweighted UniFrac PCoA plots showing subjects with short duration illness (≤30 days) before (n=19), during (n=48), and early after (n=51) infection; moderate symptom subjects before (n=14), during (n=23), and early after (n=27) infection. **B.** Inter-subject weighted and unweighted UniFrac distances within all short or long illness duration cases comparing before, during, or early after infection. **C.** Intra-subject distances (distance between timepoints for individual subjects) broken up by illness duration. Mann-Whitney U test p-values: * $p < 0.05$, ** $p < 0.01$, *** $p < 0.001$

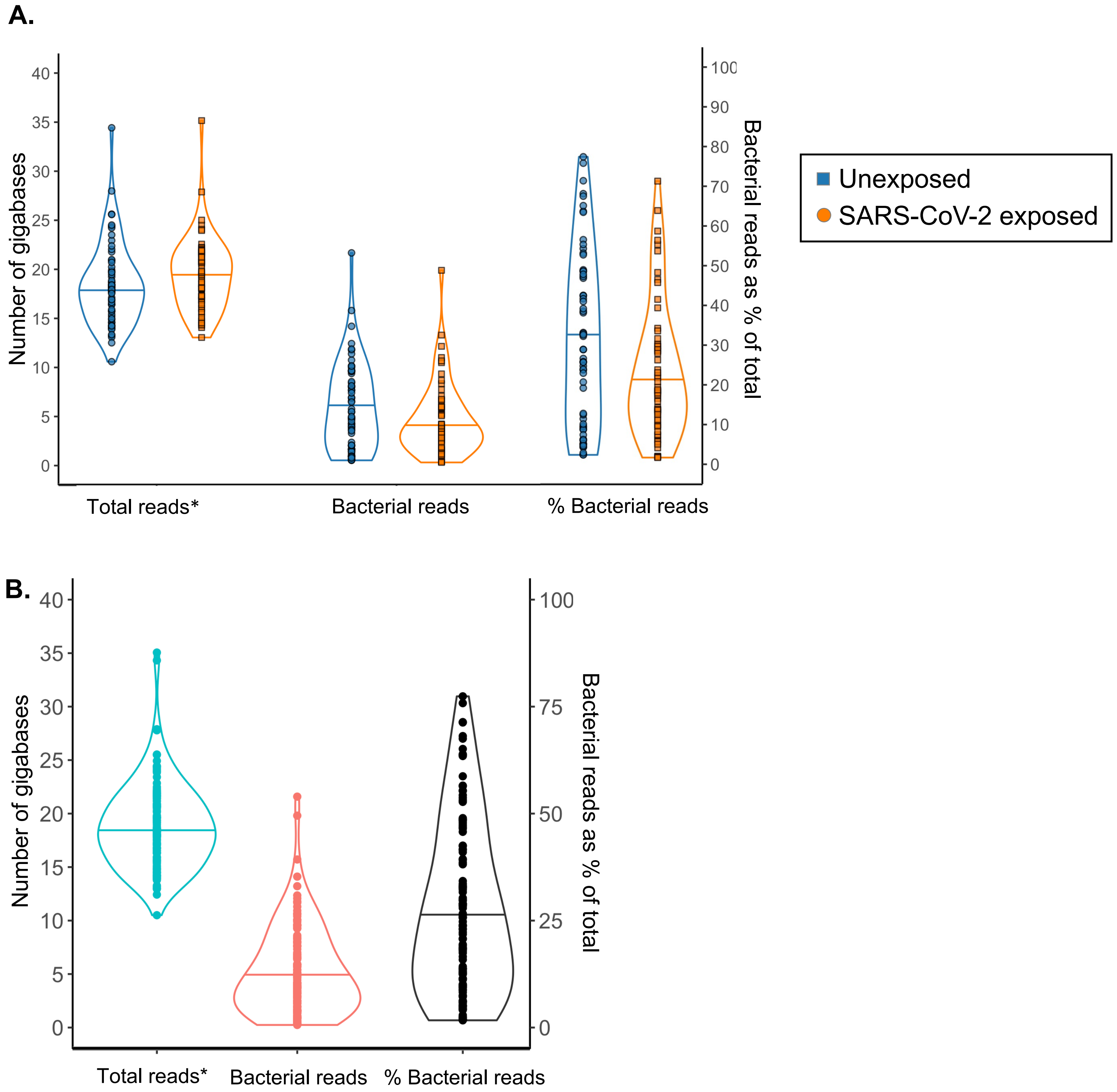

Figure S6. Summary of shotgun metagenomics sequencing of 123 samples. Summary of gigabases (GB) per sample with the total number of reads (Total reads), the reads mapping to known bacterial sequencings (Bacterial reads) and the percent of the total reads that map to bacteria (% Bacterial reads). Showing **A.** All samples and **B.** Samples broken up by SARS-CoV-2-infected cases (n=81; orange) and controls (n=57; blue). There are no significant differences between cases and controls ($p > 0.05$, Mann-Whitney U test). *One outlier sample in the control group with total reads of 84 GB not visualized but included in statistics.

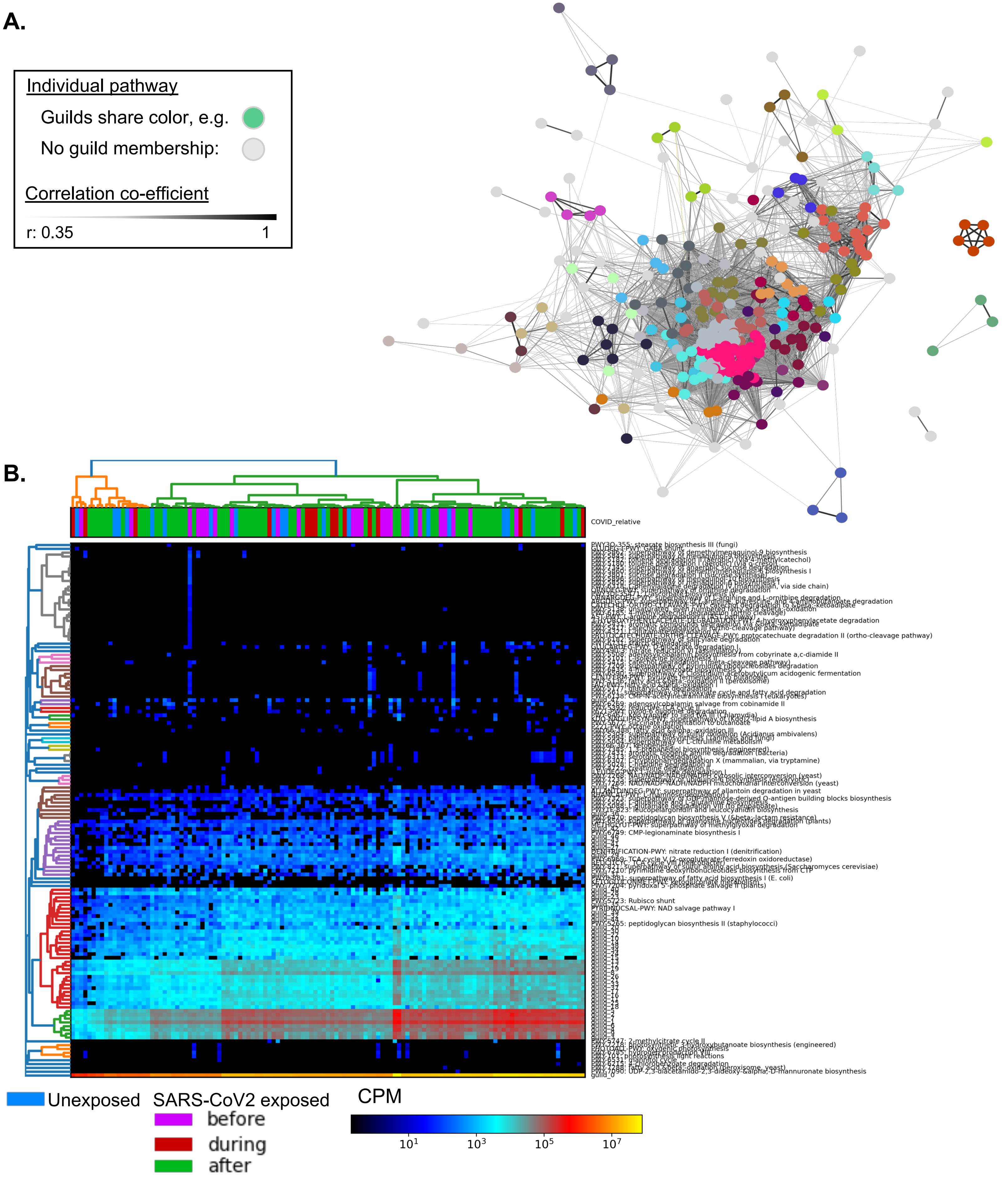

Figure S7. Co-occurrence of shotgun functional gene pathways measured in the analyzed cohort of 32 cases and 30 controls. **A.** Visualization of the co-occurence network created using Sparse Correlation Network Investigation for Compositional Data (SCNIC). Nodes represent functional pathways, and connections represent correlations between pathways. Only displayed are the pathways that have $\geq$ 1 correlation with r value > 0.35. **B.** Heatmap of the functional metagenomic pathways, shown as individual features or binned in co-occurrence guilds. Abundance of features reported as counts per million reads (CPM). Heatmap clustering of features and samples is unsupervised.

Supplemental Material S1. Statistical comparison of comorbidities between exposed and unexposed groups

```
 univar age1 gfr bmi if _m == 3, by(any_positive_match)

-> any_positive_match=0
                                        -------------- Quantiles --------------
Variable       n     Mean     S.D.      Min      .25      Mdn      .75      Max
-------------------------------------------------------------------------------
    age1      56    41.86    12.94    23.00    31.00    38.50    53.00    77.00
     gfr      56   104.80    18.76    52.64    92.53   107.33   116.26   143.75
     bmi      56    27.37     5.17    16.43    23.31    27.23    31.50    42.04
-------------------------------------------------------------------------------

-> any_positive_match=1
                                        -------------- Quantiles --------------
Variable       n     Mean     S.D.      Min      .25      Mdn      .75      Max
-------------------------------------------------------------------------------
    age1      82    41.09    12.84    23.00    31.00    36.00    51.00    76.00
     gfr      82    99.97    18.22    46.23    87.63   100.44   115.45   140.77
     bmi      82    27.49     6.87     6.14    23.29    27.63    31.66    43.77
-------------------------------------------------------------------------------

Two-sample Wilcoxon rank-sum (Mann-Whitney) test

any_positi~h |      Obs    Rank sum    Expected
-------------+---------------------------------
           0 |       56        3997        3892
           1 |       82        5594        5699
-------------+---------------------------------
    Combined |      138        9591        9591

Unadjusted variance    53190.67
Adjustment for ties      -80.40
                     ----------
Adjusted variance      53110.27

H0: age1(any_po~h==0) = age1(any_po~h==1)
         z =  0.456
Prob > |z| = 0.6487
Exact prob = 0.6505

Two-sample Wilcoxon rank-sum (Mann-Whitney) test

any_positi~h |      Obs    Rank sum    Expected
-------------+---------------------------------
           0 |       56        4187        3892
           1 |       82        5404        5699
-------------+---------------------------------
    Combined |      138        9591        9591

Unadjusted variance    53190.67
Adjustment for ties       -5.22
                     ----------
Adjusted variance      53185.44

H0: gfr(any_po~h==0) = gfr(any_po~h==1)
         z =  1.279
Prob > |z| = 0.2008
Exact prob = 0.2019

Two-sample Wilcoxon rank-sum (Mann-Whitney) test
```

```
any_positi~h |      Obs    Rank sum    Expected
-------------+---------------------------------
           0 |       56        3848        3892
           1 |       82        5743        5699
-------------+---------------------------------
    Combined |      138        9591        9591

Unadjusted variance    53190.67
Adjustment for ties       -2.31
                     ----------
Adjusted variance      53188.36

H0: bmi(any_po~h==0) = bmi(any_po~h==1)
         z = -0.191
Prob > |z| = 0.8487
Exact prob = 0.8500

. tab gender any_positive_match if _m == 3, col exact

+-------------------+
| Key               |
|-------------------|
|     frequency     |
| column percentage |
+-------------------+

                |     any_positive
         Gender |         0          1 |     Total
----------------+----------------------+----------
         Female |        40         61 |       101 
                |     71.43      74.39 |     73.19 
----------------+----------------------+----------
           Male |        16         21 |        37 
                |     28.57      25.61 |     26.81 
----------------+----------------------+----------
          Total |        56         82 |       138 
                |    100.00     100.00 |    100.00 

           Fisher's exact =                 0.701
   1-sided Fisher's exact =                 0.423

. tab race_eth any_positive_match if _m == 3, col exact

+-------------------+
| Key               |
|-------------------|
|     frequency     |
| column percentage |
+-------------------+

Enumerating sample-space combinations:
stage 5:  enumerations = 1
stage 4:  enumerations = 3
stage 3:  enumerations = 7
stage 2:  enumerations = 14
stage 1:  enumerations = 0

  Race and |
  hispanic |     any_positive
 ethnicity |         0          1 |     Total
-----------+----------------------+----------
         1 |        22         36 |        58 
           |     40.00      44.44 |     42.65 
```

```
-----------+----------------------+----------
         2 |         8         10 |        18 
           |     14.55      12.35 |     13.24 
-----------+----------------------+----------
         3 |         9         13 |        22 
           |     16.36      16.05 |     16.18 
-----------+----------------------+----------
         4 |        11         17 |        28 
           |     20.00      20.99 |     20.59 
-----------+----------------------+----------
         5 |         5          5 |        10 
           |      9.09       6.17 |      7.35 
-----------+----------------------+----------
     Total |        55         81 |       136 
           |    100.00     100.00 |    100.00 

           Fisher's exact =                 0.951

. tab hc_role_alt any_positive_match if _m == 3, col exact

+-------------------+
| Key               |
|-------------------|
|     frequency     |
| column percentage |
+-------------------+

 RECODE of |     any_positive
   hc_role |         0          1 |     Total
-----------+----------------------+----------
         0 |         8         10 |        18 
           |     14.29      12.20 |     13.04 
-----------+----------------------+----------
         1 |        48         72 |       120 
           |     85.71      87.80 |     86.96 
-----------+----------------------+----------
     Total |        56         82 |       138 
           |    100.00     100.00 |    100.00 

           Fisher's exact =                 0.799
   1-sided Fisher's exact =                 0.455

. tab hc_role any_positive_match if _m == 3, col exact

+-------------------+
| Key               |
|-------------------|
|     frequency     |
| column percentage |
+-------------------+

Enumerating sample-space combinations:
stage 5:  enumerations = 1
stage 4:  enumerations = 5
stage 3:  enumerations = 13
stage 2:  enumerations = 59
stage 1:  enumerations = 0

                      |     any_positive
              hc_role |         0          1 |     Total
----------------------+----------------------+----------
Non-healthcare worker |         8         10 |        18 
                      |     14.29      12.20 |     13.04 
```

```
----------------------+----------------------+----------
  Attending physician |         8          7 |        15 
                      |     14.29       8.54 |     10.87 
----------------------+----------------------+----------
Resident or fellow ph |         7          8 |        15 
                      |     12.50       9.76 |     10.87 
----------------------+----------------------+----------
                Nurse |        22         42 |        64 
                      |     39.29      51.22 |     46.38 
----------------------+----------------------+----------
                Other |        11         15 |        26 
                      |     19.64      18.29 |     18.84 
----------------------+----------------------+----------
                Total |        56         82 |       138 
                      |    100.00     100.00 |    100.00 

           Fisher's exact =                 0.644

. tab dm any_positive_match if _m == 3, col exact

+-------------------+
| Key               |
|-------------------|
|     frequency     |
| column percentage |
+-------------------+

  Patient has |
     Diabetes |     any_positive
     Mellitus |         0          1 |     Total
--------------+----------------------+----------
           No |        50         80 |       130 
              |     90.91      97.56 |     94.89 
--------------+----------------------+----------
          Yes |         5          2 |         7 
              |      9.09       2.44 |      5.11 
--------------+----------------------+----------
        Total |        55         82 |       137 
              |    100.00     100.00 |    100.00 

           Fisher's exact =                 0.117
   1-sided Fisher's exact =                 0.092

. tab cvd any_positive_match if _m == 3, col exact

+-------------------+
| Key               |
|-------------------|
|     frequency     |
| column percentage |
+-------------------+

           |     any_positive
       cvd |         0          1 |     Total
-----------+----------------------+----------
        No |        54         77 |       131 
           |     98.18      97.47 |     97.76 
-----------+----------------------+----------
       Yes |         1          2 |         3 
           |      1.82       2.53 |      2.24 
-----------+----------------------+----------
     Total |        55         79 |       134 
           |    100.00     100.00 |    100.00 
```

```
           Fisher's exact =                 1.000
   1-sided Fisher's exact =                 0.634

. tab bmi_cat any_positive_match if _m == 3, col exact

+-------------------+
| Key               |
|-------------------|
|     frequency     |
| column percentage |
+-------------------+

Enumerating sample-space combinations:
stage 4:  enumerations = 1
stage 3:  enumerations = 1
stage 2:  enumerations = 2
stage 1:  enumerations = 0

              |     any_positive
      bmi_cat |         0          1 |     Total
--------------+----------------------+----------
    BMI <18.5 |         1          3 |         4 
              |      1.79       3.66 |      2.90 
--------------+----------------------+----------
BMI 18.5-24.9 |        18         27 |        45 
              |     32.14      32.93 |     32.61 
--------------+----------------------+----------
  BMI 25-29.9 |        18         25 |        43 
              |     32.14      30.49 |     31.16 
--------------+----------------------+----------
      BMI ≥30 |        19         27 |        46 
              |     33.93      32.93 |     33.33 
--------------+----------------------+----------
        Total |        56         82 |       138 
              |    100.00     100.00 |    100.00 

           Fisher's exact =                 0.974
```
